# Insights into the Antimicrobial Activities and Metabolomes of *Aquimarina* (*Flavobacteriaceae*, *Bacteroidetes*) Species from the Rare Marine Biosphere

**DOI:** 10.3390/md20070423

**Published:** 2022-06-28

**Authors:** Sandra Godinho Silva, Patrícia Paula, José Paulo da Silva, Dalila Mil-Homens, Miguel Cacho Teixeira, Arsénio Mendes Fialho, Rodrigo Costa, Tina Keller-Costa

**Affiliations:** 1 IBB—Institute for Bioengineering and Biosciences and i4HB—Institute for Health and Bioeconomy, Instituto Superior Técnico, Av. Rovisco Pais, 1049-001 Lisbon, Portugal; sandragodinhosilva@tecnico.ulisboa.pt (S.G.S.); patricialcpaula@gmail.com (P.P.); dalilamil-homens@tecnico.ulisboa.pt (D.M.-H.); mnpct@tecnico.ulisboa.pt (M.C.T.); afialho@tecnico.ulisboa.pt (A.M.F.); 2 Bioengeneering Department, Instituto Superior Técnico, Av. Rovisco Pais, 1049-001 Lisbon, Portugal; 3 Centre of Marine Sciences, University of Algarve, Campus de Gambelas, 8005-139 Faro, Portugal; jpsilva@ualg.pt

**Keywords:** bioactivity screening, biosynthetic gene clusters, metabolic networks, microbial diversity, natural products, the Sponge Microbiome Project

## Abstract

Two novel natural products, the polyketide cuniculene and the peptide antibiotic aquimarin, were recently discovered from the marine bacterial genus *Aquimarina*. However, the diversity of the secondary metabolite biosynthetic gene clusters (SM-BGCs) in *Aquimarina* genomes indicates a far greater biosynthetic potential. In this study, nine representative *Aquimarina* strains were tested for antimicrobial activity against diverse human-pathogenic and marine microorganisms and subjected to metabolomic and genomic profiling. We found an inhibitory activity of most *Aquimarina* strains against *Candida glabrata* and marine *Vibrio* and *Alphaproteobacteria* species. *Aquimarina* sp. Aq135 and *Aquimarina muelleri* crude extracts showed particularly promising antimicrobial activities, amongst others against methicillin-resistant *Staphylococcus* *aureus*. The metabolomic and functional genomic profiles of *Aquimarina* spp. followed similar patterns and were shaped by phylogeny. SM-BGC and metabolomics networks suggest the presence of novel polyketides and peptides, including cyclic depsipeptide-related compounds. Moreover, exploration of the ‘Sponge Microbiome Project’ dataset revealed that *Aquimarina* spp. possess low-abundance distributions worldwide across multiple marine biotopes. Our study emphasizes the relevance of this member of the microbial rare biosphere as a promising source of novel natural products. We predict that future metabologenomics studies of *Aquimarina* species will expand the spectrum of known secondary metabolites and bioactivities from marine ecosystems.

## 1. Introduction

The largest pool of biodiversity on Earth is encapsulated in the microbial rare biosphere, the collection of microbial taxa that is present in a sample at a specific time point with a so-far arbitrarily defined relative population size of <0.1% [1]. Regardless of the system being studied, bacterial cultivation efforts tend to disproportionally favor the isolation of low-abundance populations on culture media [2,3,4,5]. Yet, cultivable members of the rare biosphere may share metabolic features with the dominant (but often uncultivable so far) members of a certain system, as has been recently suggested for the marine sponge microbiome [5]. It follows that the dedicated cultivation of rare biosphere microorganisms is a possible route for the biotechnological exploration of a significant portion of the microbiome, increasing the chances of profiting from the “genomic and metabolic reservoirs” of ecosystems [6]. From a biotechnological point of view, this genomic reservoir is a promising source of novel enzymes and metabolites. One striking example is the marine bacterial genus *Salinispora* (class *Actinomycetia*), a member of the rare biosphere and a rich source of secondary metabolites [7,8]. Nevertheless, the number of studies that have explicitly addressed the secondary metabolism of rare biosphere microbes is still reduced.

*Aquimarina* (*Bacteroidetes* phylum, *Flavobacteriaceae* family) [9] is a marine bacterial genus whose members have been isolated not only from diverse marine hosts such as sponges [10,11], algae [12,13] and octocorals [14] but also from non-host environments such as seawater [15,16,17] and sediments [18,19]. *Aquimarina* species have been earlier presumed to correspond to low-abundance populations within the marine sponge microbiome [2], but the knowledge of their large-scale abundance distributions across marine biotopes is lacking. Moreover, recent research has highlighted the potential of the *Aquimarina* genus as a source of novel bioactive compounds. For example, the activity against human-pathogenic *Staphylococcus aureus* [20], including methicillin-resistant *Staphylococcus aureus* (MRSA) phylotypes [21], has been reported for some *Aquimarina* strains. In addition, two novel compounds were recently isolated and characterized from *Aquimarina* strains derived from marine sponges: the polyketide cuniculene, isolated from *Aquimarina* sp. Aq78 [22], and the non-ribosomal peptides aquimarins, from *Aquimarina* sp. Aq135 [23]. While cuniculene bioactivities still need to be disclosed, the bioactivity of aquimarins against several human-pathogenic bacteria such as *Mycobacterium tuberculosis* and MRSA has already been reported [23]. However, the high number and diversity of secondary metabolite biosynthetic gene clusters (SM-BGCs) in *Aquimarina* genomes point towards a greater, yet to be explored, secondary metabolite production capacity [24]. Altogether, these discoveries validate the interest in *Aquimarina* spp. for the detection of new chemical compounds and point toward the suitability of this genus to marine drugs research. 

In this study, we examined the relative abundance of *Aquimarina* spp. across different marine biotopes by exploring the latest dataset release of the Sponge Microbiome Project (SMP) [25], an international effort that comprehensively mapped sponge-associated, seawater and sediment prokaryotic communities based on the next-generation sequencing of 16S rRNA genes. An analysis of this dataset allowed us to infer whether *Aquimarina* spp. are likely members of the marine microbial rare biosphere. We further assessed the antimicrobial activity of a panel of nine *Aquimarina* isolates against a broad range of human-pathogenic microbes and marine bacteria. The nine isolates represent at least six *Aquimarina* species and all major phylogenomic branches within the genus, as delineated earlier by Silva et al. [20]. They include strains isolated from the marine sponges *Sarcotragus spinosulus* (Aq78, Aq107 and Aq349 [20]) and *Ircinia variabilis* (Aq135 [20]) and the octocoral *Eunicella labiata* (EL33 and EL43 [4]), along with the type strains of the species *A*. *spongiae* (from the marine sponge *Halichondria oshoro* [10]), *A*. *muelleri* [9] and *A*. *latercula* [26] from seawater. Finally, we established reference metabolomic profiles of extracts from culture supernatants of these *Aquimarina* isolates using liquid chromatography coupled with high-resolution mass spectrometry (UPLC-HR-MS). The obtained metabolomes were used to explore possible correlations with the strain phylogeny, antimicrobial activities, and functional genome (including SM-BGC) profiles.

## 2. Results

### 2.1. Abundance Distributions of Aquimarina spp. across Marine Biotopes

**Figure 1:**
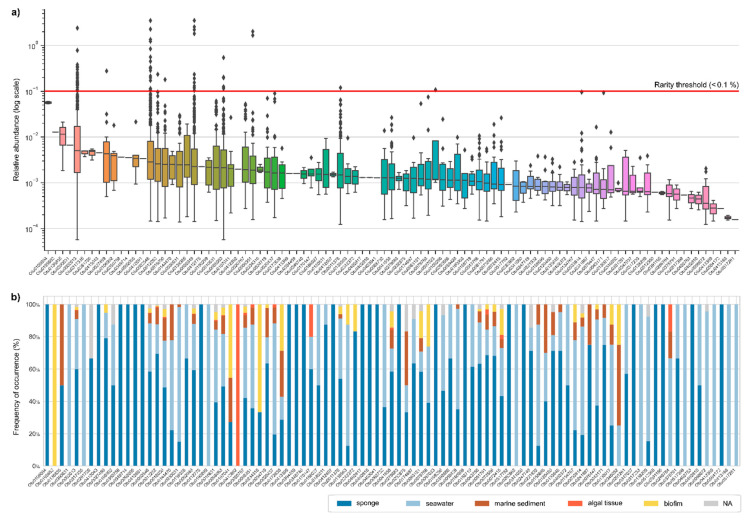
Abundance and distribution of *Aquimarina* spp. across marine habitats. (**a**) Relative abundance of each *Aquimarina* operational taxonomic unit (OTU) in the Sponge Microbiome Project (SMP) dataset. The 95 OTUs are ordered from left to right in descending order of the median relative abundance across the dataset. To facilitate visualization, the *y*-axis is presented on a logarithmic scale. The boxes represent the limits of the second and third quartiles, and the line crossing each box represents the median. Outliers are represented by a rhombus. A rarity threshold of 0.1% is represented with a red line. Notably, the median relative abundances of all OTUs except one fall below 0.01%. The color gradient of the boxes is for aesthetic purpose only. (**b**) The provenance of each OTU is displayed as the frequency of occurrence (%) of each OTU across the different habitats surveyed in the SMP dataset.

To examine the relative abundance of *Aquimarina* spp. in the marine environment, the SMP dataset was explored. We analyzed 16S rRNA gene amplicon sequencing results of 3413 marine samples after filtering out SMP samples with less than 10,000 reads. Overall, 95 operational taxonomic units (OTUs, defined at >97% gene sequence homology, a proxy for bacterial phylotypes or species) were identified as *Aquimarina,* and 535 samples (15.67%) had at least one OTU assigned through this classification. All OTUs had a mean relative abundance per sample below the defined rarity threshold of 0.1% in this study, and all except one OTU displayed median relative abundance values below 0.01% (Figure 1a). Ten OTUs, however, were above the 0.1% threshold in at least one sample (Figure 1a), suggesting that they may represent conditionally rare taxa [1,6]. Within this group, two OTUs stood out for their high frequency of occurrence across the dataset: OTU0002546, present in 471 samples, and OTU0002013, present in 466 samples (Figure S1). Nevertheless, many OTUs (60 OTUs, 63.2%) had a narrower distribution, being present in less than ten samples. Overall, the most widespread OTUs also tended to be the OTUs with a higher mean relative abundance. 

We also analyzed the OTU distributions per habitat type (Figure 1b) and found that some OTUs were exclusively present in a specific habitat. For example, nine OTUs were only identified in seawater, while one OTU (OTU0195602) was only present in algal tissue. *Aquimarina* OTUs were more often found in samples retrieved near sponges, such as marine sediments (80% of the samples had at least one *Aquimarina* OTU) and seawater (72%) than inside sponges (only 23% of these samples had at least one *Aquimarina* OTU) (Table S1). While sponge orders such as *Baerida* (5/6, 83.33%) and *Bubarida* (15/28, 53.57%) had relatively high occurrence rates of *Aquimarina* OTUs, in other orders, such as *Tetractinellida* (13/225, 5.78%), *Aquimarina* OTUs were often absent (Table S1). 

### 2.2. Diversity and Relatedness of Aquimarina OTUs 

To assess their diversity and taxonomic relatedness, the SMP-derived *Aquimarina* OTU sequences were aligned with the 16S rRNA gene sequences of all the currently known *Aquimarina* type species, as well as the nine *Aquimarina* isolates used in this study for bioactivity assessments and metabolomics (see Table S2 for more details on the isolates). A phylogenetic tree was constructed with all the sequences (Figure S2), enabling a comprehensive view of the diversity of the *Aquimarina* spp. In addition, taxonomical reclassification of all SMP OTUs was performed with the latest version of the curated Ribosomal Database Project (RDP) taxonomy database [27] and confidence values regarding their classification as *Aquimarina* were recorded (Figure S2). From the 95 OTUS in the analysis, only 14 OTUs had a confidence value above 80% and 25 OTUs between 60% and 80%. Nevertheless, the placement of SMP OTUs in the phylogenetic tree revealed that all except one (OTU0234316) presented a closer resemblance to formally described *Aquimarina* species than to species belonging to the closest relative genus *Kordia* or to other representative genera in the *Flavobacteriaceae* family (Figure S2). Additionally, several tree branches composed exclusively by *Aquimarina* SMP OTUs could be depicted from the tree, suggesting that additional phylogenetic diversity within the genus is yet to be captured by the continued cultivation efforts. Finally, we identified SMP OTUs showing a close resemblance to the cultured type strains, some of which can be employed as proxies for the *Aquimarina* strains used in this study. That was the case of OTU0002013, which was phylogenetically close to strains Aq349, EL43 and EL33, as well as to *A. megaterium* and *A. atlantica* (Figure S2) and assigned to the genus *Aquimarina* with high confidence (91%). Similarly, OTU012282 clustered closely together with the *A*. *latercula*-type strain DSM 2041 analyzed in this study (Figure S2). 

### 2.3. Antimicrobial Activities of Aquimarina spp.

**Figure 2:**
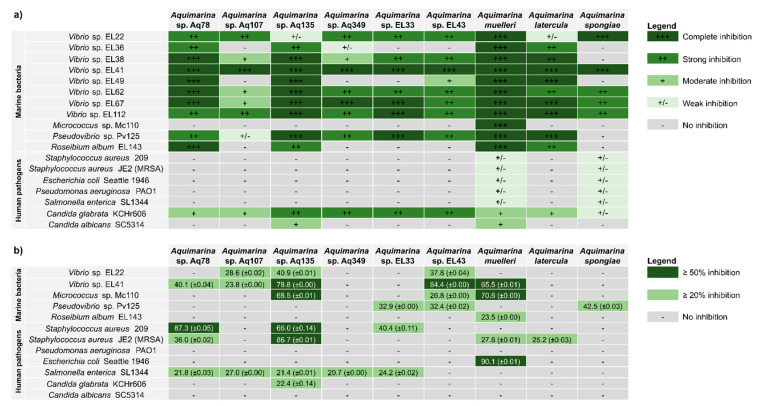
Antimicrobial activity of the *Aquimarina* strains against human pathogens and marine bacteria. (**a**) Cross-streak agar plate assay results. Inhibition of the growth of the test strains in the presence of *Aquimarina* spp. was assessed qualitatively in a screening-based fashion and ranked as: no inhibition (−), weak inhibition (+/−), moderate inhibition (+), strong inhibition (++) and total inhibition of growth compared with the negative controls without *Aquimarina*. Each *Aquimarina* test strain pair was at least tested twice. (**b**) Broth microdilution assay results with the *Aquimarina* crude extracts. Percentage of the inhibition of the test strain growth by each extract was calculated as $\frac{OD\;control-OD\;extract}{OD\;control}\times 100$. Average percentages (and their respective standard deviations) of the growth inhibition of the test strains in the presence of 10% *v*/*v Aquimarina* crude extracts compared with the controls (i.e., the test strain grown in the absence of extracts) are shown. Values correspond to two replicates.

The antimicrobial activities of nine *Aquimarina* strains against a panel of eleven marine bacteria and seven human-pathogenic microorganisms (Figure 2a and Tables S3 and S4) were assessed using the cross-streak method, a relatively simple and fast screening method that allows the testing of large numbers of isolates from a given culture collection for antimicrobial activity against a wide range of microorganisms [28]. *Aquimarina* isolates displayed consistent inhibitory activity against most of the tested marine bacteria, particularly against Gram-negative *Vibrio* spp. Only one marine test strain, Gram-positive *Micrococcus* sp. Mc110 (*Actinobacteria*), was not inhibited by eight out of nine *Aquimarina* strains in these assays. In contrast, the complete inhibition of *Vibrio* sp. EL41 (*Gammaproteobacteria*), whose closest type species is *Vibrio breoganii* (Table S4), was observed for all *Aquimarina* isolates (Figure 2a). *A. muelleri* was the *Aquimarina* strain that was most active against marine bacteria, resulting in the complete inhibition of all marine isolates. In contrast, only a weak inhibition, by *A*. *muelleri* and *A*. *spongiae,* was found against human-pathogenic bacteria. However, the human-pathogenic yeast *Candida glabrata* KCHr606 was inhibited by all *Aquimarina* strains, and *C. albicans* SC5314 was inhibited by Aq135 and *A. muelleri* (Figure 2a and Figure S3). 

The antimicrobial activities observed in the cross-streak assays prompted us to prepare metabolite extracts from the *Aquimarina* spp. to further investigate their bioactivities. Extracellular metabolite extracts (here referred to as ‘crude extracts’) were prepared by solid-phase extraction (SPE) from the culture supernatants of all *Aquimarina* isolates and tested against the seven human microbial pathogens and five representative marine bacteria (*Vibrio* sp. EL22 and EL44, *Micrococcus* sp. Mc110, *Pseudovibrio* sp. Pv125 and *Roseibium album* EL143) (Figure 2b) using broth microdilution assays. Gram-positive human pathogens, represented by *S. aureus* strain 209 and MRSA strain JE2, were strongly inhibited (≥50% growth reduction) by the *Aquimarina* sp. Aq135 extract. Moderate growth inhibition (20% ≥ I < 50%) of MRSA was observed for *Aquimarina* sp. Aq78, *A. muelleri* and *A. latercula* extracts. Growth of the Gram-positive marine *Micrococcus* sp. Mc110 was strongly reduced (≥50%) by *A. muelleri* and Aq135 extracts. Strong inhibition of Gram-negative *Escherichia coli* strain Seattle 1946 was observed for the *A. muelleri* extract, while moderate inhibition of *Salmonella enterica* strain SL1344 was triggered by extracellular extracts of strains Aq78, Aq107, Aq. 135, Aq349 and EL33. Finally, moderate growth inhibition of *C. glabrata* growth was observed with the Aq135 extract.

**Figure 3:**
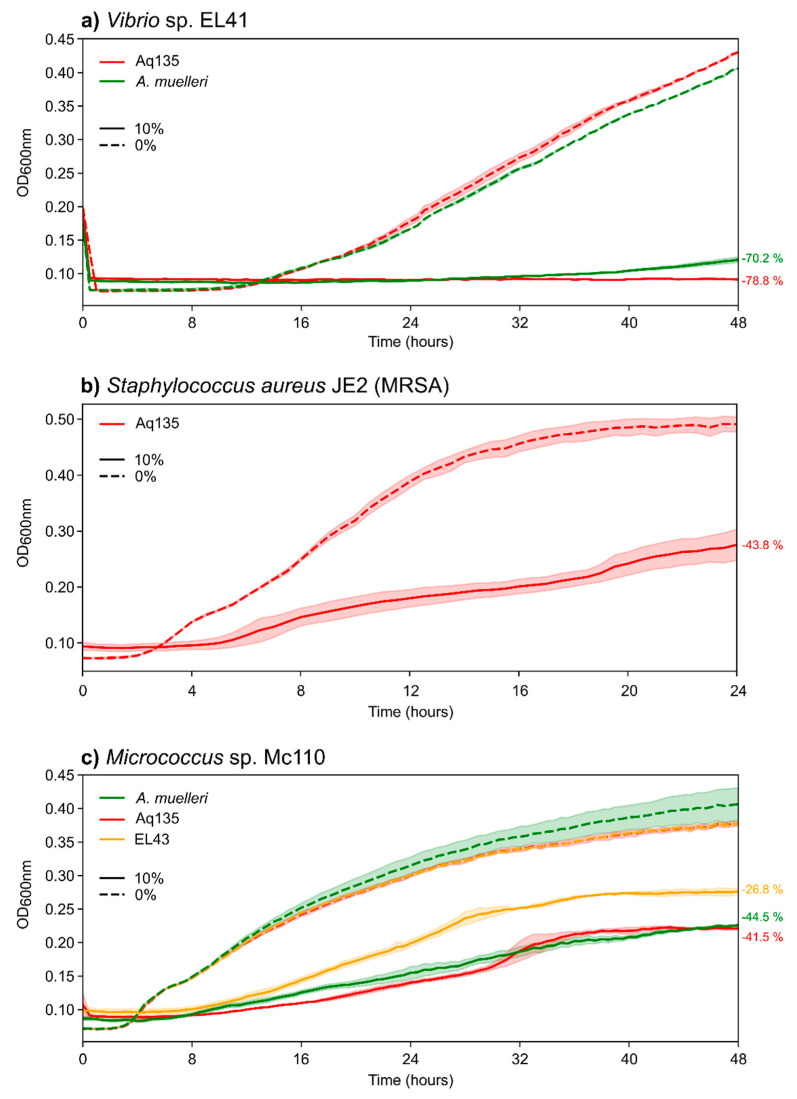
Growth curves of the test strains in the presence of *Aquimarina* crude extracts at 10% *v*/*v* concentration (full lines) versus the absence of *Aquimarina* extracts (0% *v*/*v*; dashed lines). Red lines represent the Aq135 extract, green lines the *A*. *muelleri* extract and the yellow line the EL43 extract. Each *Aquimarina* crude extract was tested twice on each test strain, and lines represent the average OD_600 nm_ values, while shades behind lines represent 95% confidence intervals. Percentages of growth reduction relative to the control are displayed next to the respective curves on the right.

To study test strain responses in more detail (Figure 3), we also generated growth curves for sensitive test strains in the presence of the most potent *Aquimarina* extracts. We observed 78.8% and 70.2% growth reduction (compared to the controls) and a significantly prolonged lag phase in *Vibrio* sp. EL41 in the presence of Aq135 and *A. muelleri* extracts, respectively (Figure 3a). Prolongation of the lag phase and a less pronounced exponential phase, together with an overall flatter curve and reduced growth, were also observed for MRSA strain JE2 in the presence of the Aq135 extract (Figure 3b) and for *Micrococcus* sp. Mc110 in the presence of the Aq135, *A. muelleri* and EL43 extracts (Figure 3c).

Overall, the inhibitory activity observed in the cross-streak plate assays varied slightly from the broth microdilution assays. For example, the extracellular extract of *Aquimarina* sp. Aq349 could not reproduce inhibitory activity against several *Vibrio* strains, *Pseudovibrio* sp. Pv125 and *C. glabrata* but presented activity against *Salmonella enterica*. Generally, in the cross-streak assays, the *Aquimarina* spp. presence provoked a much stronger growth inhibition of marine bacteria than of human pathogens, while the broth microdilution assays with extracellular *Aquimarina* crude extracts revealed some promising inhibitory activities against human-pathogenic bacteria.

### 2.4. Liquid Chromatography-Mass Spectrometry (LC-MS)-Based Metabolomics Analysis of Aquimarina Extracts

An untargeted UPLC-HR-MS/MS approach was used to explore the metabolite profiles of the extracellular *Aquimarina* extracts obtained by SPE. Extracted ion chromatograms (EIC) from full scan measurements showed a high similarity between biological replicates (three independent extracts were analyzed from each strain), indicating a high reproducibility, and reduced biological variations between replicate extracts (Figures S4 and S5).

**Figure 4:**
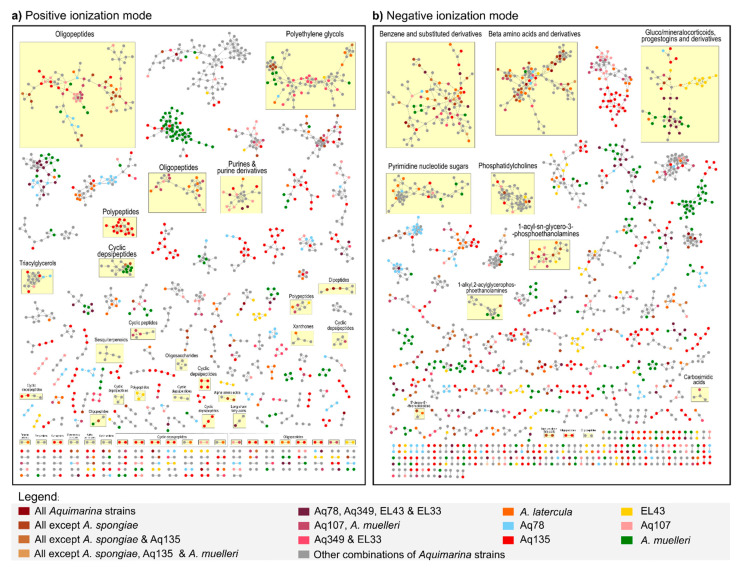
Molecular network analysis of *Aquimarina* spp. metabolome profiles. Classical (MS/MS) molecular networking of the UPLC-HR-MS/MS analysis of the (SPE) crude extracts obtained from culture supernatants of the nine *Aquimarina* strains under study. The molecular networks were constructed in GNPS and visualized in Cytoscape with an unweighted ‘Prefuse Force Directed Layout’. The results shown here already contemplate the blank (i.e., ‘culture medium-only’ controls) peak removal. Each node represents the MS/MS consensus spectrum for a certain parent mass (compound), and the connection between nodes (light grey lines) represents a high similarity between the compound spectra. Only clusters with two or more compounds are displayed (singletons were excluded from the visualization). The nodes are color-coded based on the *Aquimarina* strain (or a combination of several strains) they were identified from. Light yellow boxes highlight the chemical compound classes annotated with the MolNetEnhancer workflow (ClassyFire algorithm).

To visualize connections and correspondences between the metabolomic profiles from the nine *Aquimarina* strains, we performed classical molecular networking in the Global Natural Product Social Molecular Networking (GNPS) environment [29] and subsequent metabolite annotation using the MolNetEnhancer tool [30]. For this analysis, the metabolites present in the blank samples (i.e., extracts prepared from ‘culture medium-only’ supernatants) were removed from all *Aquimarina* samples. Both ionization modes had a similar number of nodes in the molecular network (positive ion mode: 2801; negative ion mode: 2874) (Figure 4), whereby each node represented the MS/MS consensus spectrum for a certain parent mass. From these, 2282 nodes (81%) and 2478 nodes (86%) were left unclassified in the positive and negative ion modes, respectively, pointing to a small number of nodes that could be dereplicated as known metabolites.

The number of metabolites simultaneously identified in all *Aquimarina* strains was small: 16 in the positive ion mode and 15 in the negative ion mode. However, these numbers increased to 78 and 94, respectively, when *A. spongiae* DSM 22623 was not considered, thus pointing to a distinct metabolome profile of this strain. Many nodes in the molecular networks were strain-specific (i.e., only found in one strain): 1414 in the positive and 1372 in the negative ion mode. The *Aquimarina* isolate with more strain-specific nodes was Aq135, with 431 in the positive and 390 in the negative ion mode. In addition, various classified and unclassified clusters almost exclusively composed of Aq135-specific nodes were detected in both molecular networks. One such example was an Aq135-specific polypeptide cluster (Figure 4a).

In the positive ionization mode, 31 peptide-related clusters were identified: 11 oligopeptide clusters (with 163 nodes in total), 14 cyclic depsipeptide clusters (113 nodes), 3 polypeptide clusters (46 nodes), 2 cyclic peptide clusters (21 nodes) and 1 dipeptide cluster (7 nodes). In addition, a large cluster (79 nodes), classified as polyethylene glycols, was observed with many nodes derived from strains Aq349 and EL33. Lipid-like compound classes, such as sesquiterpenoids, triacylglycerols and long-chain fatty acids, were further annotated in the positive ion mode. However, the total number of lipid-like nodes was larger in the negative (136) than in the positive (34) ion mode. Eighty of these nodes were identified as glycerophospholipids, including 44 phosphatidylcholine ions (Figure 4b). Moreover, 102 benzenes (and substituted derivatives); 95 beta-amino acids and derivatives; 53 gluco/mineralocorticoids, progestogins and derivatives and 47 pyrimidine nucleotide sugar-derived nodes were identified in the negative ion mode.

**Figure 5:**
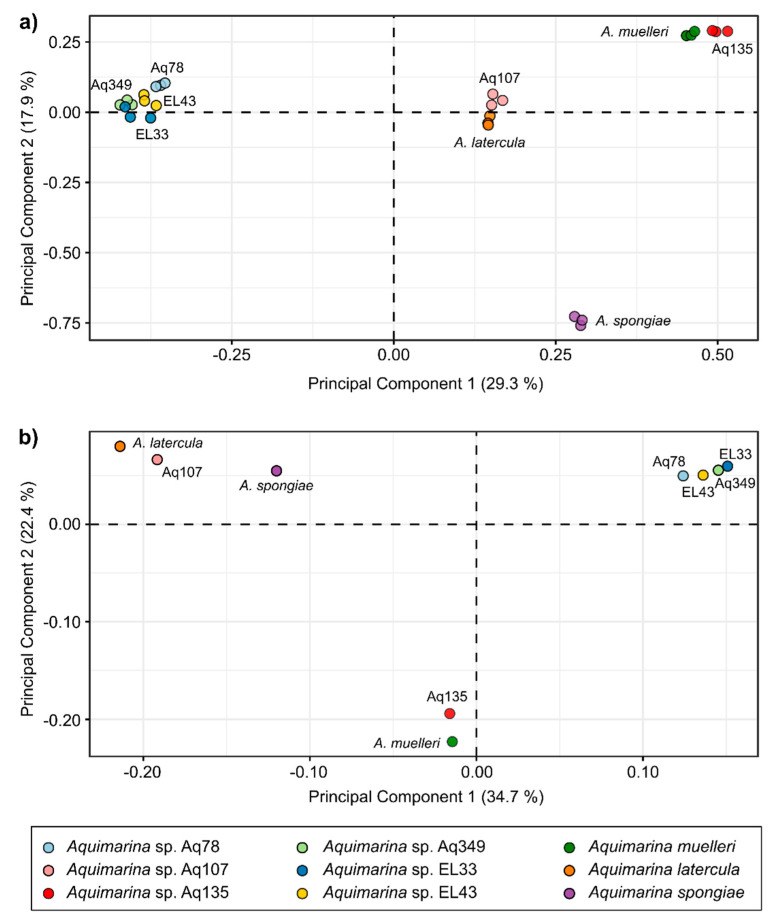
Multivariate analyses of metabolomic (**a**) and functional genomic (**b**) profiles of *Aquimarina* spp. Principal components analyses (PCA) were performed using the Euclidean distance matrix calculated from Hellinger-transformed data of (**a**) the metabolome profiles obtained for each of the nine *Aquimarina* strains in the study and (**b**) Pfam-based annotation of the corresponding genome assemblies of the same strains. The ordinations are shown on an Eigenvalue scale. The *x*- and *y*-axes represent Principal Components 1 and 2, respectively, while the percentages in brackets indicate how much of the overall variance in each dataset is explained by each principal component. Overall, the closer the colored dots that represent the *Aquimarina* samples are to each other, the more similar are their respective metabolomics (**a**) or functional genomics (**b**) profiles. The input data for the PCA of the metabolome profiles derived from a feature-based molecular networking (FBMN) analysis with both ionization modes merged (see Table S5 for details). Chemical profiles of three SPE extracts prepared from three culture supernatants (independent replicates) were analyzed per *Aquimarina* strain.

To conduct multivariate analyses of *Aquimarinas* metabolome profiles, we also performed feature-based molecular networking (FBMN; Table S5). As in classical molecular networking, FBMN uses MS^2^ data to perform spectral clustering but also incorporates MS^1^ information such as retention time and isotope patterns in the analysis [31]. This enhances the probability of distinguishing isomers with identical MS^2^ spectra that may remain unnoticed in classical molecular networking. The principal components analysis (PCA) revealed four distinct clusters formed by *Aquimarina* strains based on their metabolite profiles: the first one comprising EL33, EL43, Aq349 and Aq78; the second one comprising Aq107 and *A. latercula*; the third one comprising Aq135 and *A. muelleri* and the fourth one comprising only the three biological replicates of *A. spongiae* (PERMANOVA: *F* = 26.06, *p* = 0.0001) (Figure 5a). Noteworthily, the FBMN analysis identified the recently discovered *trans*-AT polyketide cuniculene in all Aq78 extracts (Table S5), indicating that the workflow employed here is suitable for the recovery of *Aquimarina*-typic secondary metabolites.

We then explored if the clustering of the *Aquimarina* metabolome profiles exhibited patterns similar to the genome functional profiles of the same strains. Therefore, a second PCA was performed using protein family (Pfam) annotations of the nine *Aquimarina* genomes (Figure 5b). Indeed, the clustering of *Aquimarina* strains based on Pfam profiles followed the pattern observed in the metabolome profiles, with Aq135 clustering together with *A. muelleri*; a second tight cluster formed by EL33, EL43 Aq349 and Aq78 and a third cluster formed by Aq107 and *A. latercula* (PERMANOVA: *F* = 10.84, *p* = 0.0007), while *A. spongiae* was rather separated from the other genomes.

### 2.5. SM-BGC Identification on Aquimarina Genomes

The biosynthetic potential of each *Aquimarina* strain was further explored by annotating SM-BGCs with antiSMASH v. 6.0.1 [32] (Figure S6 and Table S6). The counts ranged from a minimum of seven SM-BGCs in strain Aq107 to a maximum of twenty-one SM-BGCs in *A. muelleri*. However, the *A. muelleri* genome had the highest percentage of incomplete SM-BGCs (62%) (Table S7). Indeed, the *A. muelleri* genome assembly was more fragmented (107 contigs), increasing the likelihood of incomplete SM-BGCs. In contrast, the PacBio sequenced genomes of Aq78, Aq107, Aq135, Aq349 and EL43, which only comprised one to three contigs, respectively, did not present any fragmented SM-BGCs on the contig edges.

**Figure 6:**
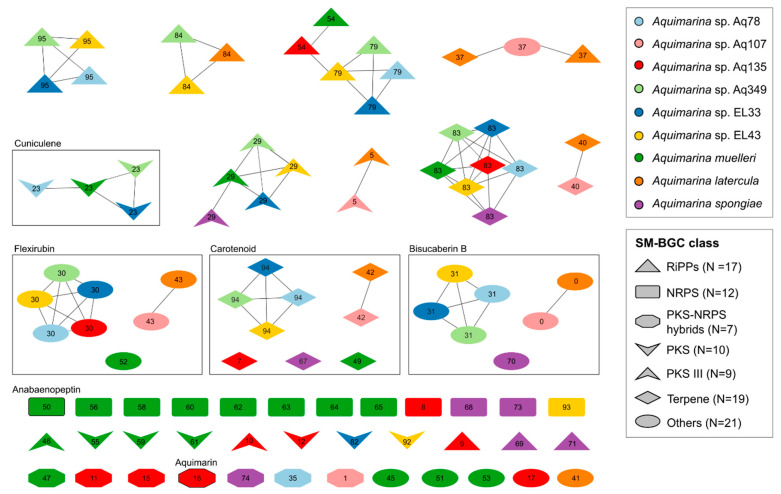
Sequence similarity network (SSN) of the *Aquimarina* secondary metabolite biosynthetic gene clusters (SM-BGCs). Each node represents a single SM-BGC, identified by antiSMASH v6.0.1, in a specific *Aquimarina* strain (color-coded). The shape of each node identifies to which compound class the SM-BGC belongs. Grey lines connecting certain SM-BGCs indicate their respective grouping into the Gene Cluster Families (GCFs) and Gene Cluster Clans (GCCs). Each GCF and singleton SM-BGC (i.e., SM-BGCs that did not cluster into any GCF) is identified by a number (ID). SM-BGCs or GCFs coding for a known compound are labeled with the respective compound name. The SSN was constructed using BiG-SCAPE with a 0.3 cut-off and visualized in Cytoscape using an unweighted ‘Prefuse Force Directed Layout’. Abbreviations: RiPPs, ribosomally synthesized and post-translationally modified peptides; NRPS, non-ribosomal peptide synthetase; PKS, polyketide synthase. The category ‘Others’ includes SM-BGCs that were less frequent on the *Aquimarina* genomes such as siderophore, resorcinol or arylpolyene SM-BGCs, or other hybrid SM-BGCs. See Figure S6 and Table S6 for details.

SM-BGC pairwise similarities were computed with the BiG-SCAPE pipeline [33], and a Sequence Similarity Network (SSN) was constructed to illustrate the biosynthetic diversity within these nine genomes (Figure 6 and Table S8). This SSN was composed of 16 Gene Cluster Families (GCFs, i.e., groups of highly similar SM-BGCs) and 40 singletons (a total of 96 SM-BGCs). Only one Gene Cluster Clan (GCC, i.e., a group of moderately similar SM-BGCs) was found, encompassing ribosomally synthesized and post-translationally modified peptide (RiPP) GCFs (54 and 79).

All *Aquimarina* genomes had at least two terpene SM-BGCs, one siderophore and one type III polyketide synthase (PKS) SM-BGC. The type III PKS SM-BGCs comprised two GCFs, one with Aq78, Aq349, EL33, EL43 and *A. spongiae* and another one with *A*. *latercula* and Aq107, in addition to Aq135 and *A. muelleri* singletons. The SM-BGCs from *A. latercula* and strain Aq107 only formed GCFs with one another and never with other *Aquimarina* strains. SM-BGCs from *A spongiae* were only present in two GCFs, namely PKS GCF 29 and terpene GCF 83.

Some SM-BGCs coded for known compounds such as bisucaberin B; flexirubin; carotenoids; anabaenopeptin (in *A. muelleri* only); the recently described cuniculene (in *A. muelleri*, Aq78, Aq349 and EL33) and aquimarin (in Aq135 only). Bisucaberin B-, flexirubin- and carotenoid-encoding SM-BGCs were identified on all genomes, except *A. spongiae*, which lacked a flexirubin SM-BGC, and *A. muelleri* and Aq135, which lacked the bisucaberin B SM-BGC.

## 3. Discussion

### 3.1. Aquimarina Is a Member of the Microbial Rare Biosphere

The exploration of the SMP dataset, which contains a comprehensive variety of *Aquimarina*- and *Aquimarina*-related OTUs, allowed us to infer, for the first time, that species of the *Aquimarina* genus are part of the rare marine biosphere. Although some evidence existed earlier [2], a formal assessment of the *Aquimarinas* relative abundances in marine ecosystems has never been performed. The SMP dataset was chosen due to its great taxonomical sponge diversity (81 sponge species) from widespread geographic locations (from subpolar to tropical waters) and for the availability of an extensive number of environmental (seawater and sediment) samples [25]. In addition, sponges are considered excellent animal models for symbiosis and marine drug research due to their rich microbiota and biochemistry [34]. Moreover, the five *Aquimarina* strains analyzed in this study have been isolated from sponges: Aq78, Aq107 and Aq349 from *Sarcotragus spinosulus*; Aq135 from *Ircinia variabilis*; both sponges from the Irciniidae family (Demospongiae class, Dictyoceratida order) [35,36] and *A. spongiae* from *Halichondria oshoro* (Demospongiae class, Suberitida order) [10].

In this study, an *Aquimarina* rarity in marine settings was predominantly confirmed by the absence of OTUs with a mean relative abundance above the rarity threshold (0.1% relative abundance). Moreover, most OTUs were present in less than ten samples, and only a small percentage of samples harbored at least one *Aquimarina* OTU (15.65%). This provides compelling evidence for a predominantly rare mode of occurrence of *Aquimarina* species across multiple marine habitats.

SMP OTUs were clustered at a 97% similarity threshold, meaning that every OTU may be considered a proxy for a different species [25]. Nevertheless, some studies suggest this cut-off to be too loose to define a species, mainly when applied to short regions, such as the V4 region of the 16S rRNA gene used here [37]. From that perspective, the 95 *Aquimarina*-related OTUs can mask a greater number of *Aquimarina* species and subspecies. Several OTUs did not closely affiliate with the known *Aquimarina*-type strains, pointing towards a wealth of likely unknown *Aquimarina* species yet to be cultivated. This hypothesis was further supported by the many recent reports of new *Aquimarina* isolates [4,16,18,21,38] and metagenome-assembled genomes (MAGs) [39,40,41], even though this genus was already described in 2005 [9]. Furthermore, an open pangenome was observed in our previous comparative genomics study, indicating that genomic redundancy was still far from being achieved [24]. For all these reasons, one can presume that there are still several *Aquimarina* species yet to be discovered, and as more marine environments are explored, the number of species will likely increase.

There is still some debate about the lifestyle of *Aquimarina* spp. and the potential roles this taxon has in host-associated settings [24]. However, evidence is mounting towards a free-living lifestyle of *Aquimarina* species coupled with commensal, opportunistic or pathogenic behaviors in host associations. In the SMP dataset analyzed in this study, *Aquimarina* was more often identified in sediments than in the sponge samples, pointing towards a lack of preference for host-associated settings. However, it is important to consider reports of *Aquimarina* species as emerging pathogens of algae [13,42] and crustaceans [38,43]. In a recent metagenomics study of the octocoral microbiome, an *Aquimarina* OTU was enriched in necrotic octocoral tissue, suggesting it could be an indicator of dysbiosis [44]. In addition, an *Aquimarina* sp. MAG was retrieved from the microbial metagenome of necrotic *Eunicella gazella* tissue with very high MAG coverage [41]. These findings suggest that *Aquimarina* is, in fact, a so-called conditionally rare taxon [1,6] with the capacity to increase in abundance at certain time points and under favorable conditions. *Aquimarina* spp. are known for their extensive carbon degrading capabilities, possibly enabling these species to colonize diverse marine niches [45]. Their genomes are also significantly larger than those of other, mainly host-associated, *Flavobacteriaceae* members (Silva et al., unpublished data), further supporting the idea that *Aquimarina* species may alternate between free-living and host-associated lifestyles and are often opportunistic colonizers of marine hosts.

Despite a few examples, such as the widely studied marine actinomycete *Salinispora* [7,8], the link between rare biosphere members and their potential to synthesize novel bioactive natural products has rarely been explored. We found OTU0002013, present in 466 samples, to be closely related with strains Aq349 (isolated from a marine sponge), EL33 and EL43 (both isolated from octocoral), which exhibited rich SM-BGC profiles and antimicrobial activities, particularly against marine *Vibrio* spp. Moreover, Aq349 is a known producer of the recently discovered polyketide cuniculene [22], and this study showed that both Aq349 and EL33 (as well as Aq78 and *A. muelleri*) possess the cuniculene SM-BGC. This adds evidence to the hypothesis that the microbial rare biosphere is a prolific, underexplored source of novel bioactivities and metabolites.

### 3.2. Aquimarina Strains Inhibit Other Marine Bacteria

Using the cross-streak assays as a first, easy-to-perform screening method [28], this study reported the antimicrobial activity of nine *Aquimarina* strains against a diversified panel of marine and human-pathogenic microorganisms. All *Aquimarina* strains showed inhibitory activity against several microorganisms, and strong activity was found against *Vibrio* spp. The *Vibrio* genus contains multiple species that may coexist in commensal relationships with several marine organisms, such as fish and crustaceans. However, under specific circumstances, they can display pathogenic behaviors, causing diseases collectively referred to as vibriosis [46,47]. Vibriosis is particularly relevant in aquaculture, where sudden disease outbreaks can lead to acute economic losses [48]. Thus, future studies should isolate and purify the underlying antibacterial compounds and investigate their chemical structures, cytotoxicity, and molecular mechanisms of action to evaluate if *Aquimarina*-derived metabolites could be applied in the combat against vibriosis. *Vibrio* sp. EL41, whose closest type strain is *Vibrio breoganii,* was the most sensitive *Vibrio* strain, both in the cross-streak and broth microdilution assays. Contrary to other *Vibrio* species, which are mostly generalists, *V. breoganii* specializes in the degradation of macroalgae substrates [49]. This specialization is accompanied by genome size reduction and the loss of multiple functional genes in this species, which might explain an enhanced sensitivity to antimicrobial compounds [49].

### 3.3. Aquimarina as a Source of Novel Inhibitory Compounds against Human-Pathogenic Bacteria and Yeast

Several *Aquimarina* extracts inhibited human-pathogenic bacteria, such as MRSA, *E. coli* and *S. enterica*, to varying extents. These results are encouraging, considering the worldwide emergence of antimicrobial-resistant pathogens and the urgent need to find novel therapeutic leads [50]. They are in line with the recent literature that reported the growth inhibition of MRSA by an *A. macrocephali* strain obtained from a marine sponge [21]. The activity against MRSA was also just reported for aquimarins, a class of peptide antibiotics isolated from Aq135 [23]. Indeed, the manual inspection of UPLC-HR-MS chromatograms from Aq135 confirmed the presence of aquimarins in the Aq135 crude extracts analyzed in this study, suggesting the contribution of these compounds to the observed inhibition of MRSA by Aq135 extracts. Moreover, our SM-BGC comparison revealed that aquimarins are encoded on a singleton, polyketide synthase–non-ribosomal peptide synthetase (PKS-NRPS) hybrid cluster exclusive of strain Aq135.

*Aquimarina* spp. also displayed inhibitory activity against fungal pathogens, here represented by two *Candida* species (*C. glabrata* and *C. albicans*) which, together, are the causal agent of more than 60% of all human candidiasis cases [51]. To the best of our knowledge, this is the first report of antifungal activity in *Aquimarina* spp. *C. glabrata*, which has a higher incidence of drug resistance [52], was more often inhibited in the cross-streak assays when compared to *C. albicans*, suggesting a higher sensitivity of *C. glabrata* to *Aquimarina*-derived molecules. Few antifungal compound classes (i.e., azoles, polyenes, echinocandins and pyrimidine analogs) are currently available for the treatment of *Candida* infections, and the last decades were accompanied by a rise in drug-resistant clinical *Candida* strains [53,54]. Hence, the urge to discover new and effective anti-*Candida* drugs is higher than ever, which is why the findings of this study are exciting and deserve further exploration.

### 3.4. Aquimarina Bioactivity Profiles Change According to Experimental Conditions

Unlike the cross-streak assay that widely lacked positive results for the inhibition of Gram-positive bacteria, we did observe inhibition against Gram-positive bacteria in the presence of crude extracts from several *Aquimarina* species in the broth microdilution assays. This points to the presence of Gram-positive inhibitor compound(s) in these extracts that are not produced when grown on a solid medium or that do not diffuse well in solid agar. Conversely, the widespread inhibition of *C. glabrata* by all *Aquimarina* strains tested in the cross-streak assays was not replicated in the broth microdilution tests, except for the Aq135 extract. Several reasons might explain the observed differences between the two assay types. First, the test strains might have different sensibilities to potential inhibitory compounds when grown in a solid versus liquid medium. Second, a compound that is not produced or that does not diffuse well in solid agar might be produced when *Aquimarina* strains are grown in liquid culture. Different incubation conditions may result in the biosynthesis of different metabolites at different concentrations and, hence, different inhibitory capacities in each assay. It is well-known indeed that, although present on an organism’s genome, SM-BGCs can be ‘silent’, i.e., they are not transcribed, and therefore, the metabolite is not produced. The ‘one strain, many compounds’ (OSMAC) approach [55] explores these ‘silent’ SM-BGCs by testing different experimental conditions to increase the probability of promoting crucial induction factors and, therefore, to stimulate the production of (desired) secondary metabolites.

Possibly, the absence of inhibition towards human-pathogenic bacteria in the cross-streak assays could also be explained by the need to implement modifications to the culture medium preparation to permit the concomitant growth of marine and non-marine organisms on the same culture plate. Non-marine organisms (i.e., human pathogens) grew poorly in Mueller–Hinton broth supplemented with 3% artificial sterile seawater (ASW). Therefore, the concentration of salt had to be decreased to 2% (see the Materials and Methods for details). Although this modification was beneficial for the growth of the test strains, it might have impacted the secondary metabolism of the *Aquimarina* strains. Differently, in broth microdilution assays where media compatibility was no longer an issue, the inhibition of human bacterial pathogens such as *S. aureus* was obtained. Our results underpin the importance of testing different experimental conditions in bioactivity screenings, as this can enlarge the observed activity spectrum of the organism under study.

However, the number of replications performed in our antimicrobial assays was small, which may be of concern, since the biological variations between replicate samples in broth microdilution and other antimicrobial plate assays were earlier reported to be rather high [56,57]. Nevertheless, our study presents a first and valid screening-based effort, unveiling antimicrobial activities for a variety of *Aquimarina* species against a diverse panel of bacterial and fungal pathogens, as well as marine microorganisms. This opens new avenues for future, in-depth explorations of *Aquimarina* natural products, particularly in sectors where they have so far been overlooked (e.g., as antifungals or in aquaculture/mariculture applications).

### 3.5. Metabolomics Sheds Light on the Unknown Aquimarina Chemical Space and Indicates Presence of Novel, Cyclic Depsipeptide-Related Compounds

To gather a global perspective of the *Aquimarina* chemical space, we performed an UPLC-HR-MS/MS analysis of the crude extracts from culture supernatants of the nine strains. In positive ionization mode, several classes of peptidic compounds, such as polypeptides, cyclic depsipeptides and oligopeptides, among others, were detected. These compounds can be the product of secondary metabolic pathways encoded on NRPS, hybrid PKS-NRPS or RiPPs clusters [58], which are indeed present in great variety on the genomes of *Aquimarina* spp. We found as many as 14 cyclic depsipeptide clusters in the *Aquimarina* metabolome, with the largest cluster comprising multiple nodes from *A. muelleri*, while six smaller clusters were specific to Aq135. Depsipeptides are known for their varied bioactivities. Some are antifungals, such as the antimycins, which are active against *Candida utilis* [59], while others have been found to possess antibacterial [60], antiviral, anticancer or immunosuppressive activities [61]. Considering that Aq135 and *A.* *muelleri* displayed the strongest and widest antimicrobial activities in our study, it is tempting to speculate that novel, cyclic depsipeptides (or structurally related compounds) might have contributed to these inhibitions, something that deserves further investigation. Future bioassay- and/or genomics-guided fractionation of the *Aquimarina* crude extracts and the isolation of compounds are indeed indispensable to discern which compound(s) and compound classes contributed most to the various antifungal and antibacterial activities observed in this study. Given the large number and variety of SM-BGCs present on *Aquimarina* genomes and the complex nature of their crude extracts, it is possible that the antimicrobial activities observed here were caused by a blend of different compounds. The coproduction of multiple, synergistically acting secondary metabolites has already been described in some *Streptomyces* species and can increase the competitiveness of the producer strain in its natural habitat [62,63]. This is likely an important evolutionary driving force for bacteria that maintain a rich and diverse secondary metabolism.

In the negative ionization mode, we frequently identified non-secondary metabolites such as amino acids, nucleotides, and their corresponding derivatives. Here, the difficulty of discerning the primary and secondary metabolisms, a global problem of metabolomics studies reveals itself. Indeed, most algorithms and tools used to classify the molecules, such as the ClassyFire tool, were originally designed for non-natural product-related analyses, and some secondary metabolite classes might go unnoticed [64,65]. Moreover, the compound identification and, consequently, dereplication of each sample is dependent on the quality of the databases in use. Even the combination of several distinct databases, as used in this study for the annotation of *Aquimarinas* metabolomes, grants access only to a fraction of nature’s chemical space [66]. In contrast with the proteomics field, where over 50% of the proteins will have functional annotations, only 2–5% of the observed compounds can currently be matched to known metabolites in a typical metabolomics dataset [66]. Here, complementary genomic information can give further important insights into the chemical potential of the strains under investigation. Indeed, inspection of the *Aquimarina* spp. genomes revealed a variety of SM-BGCs encoding for likely novel type III polyketides, ribosomal and non-ribosomal peptides, and terpenes. Moreover, we detected cuniculene in the Aq78 extracts but not in the Aq349, EL33 and *A*. *muelleri* extracts, although we found the four strains to harbor the *trans*-AT PKS SM-BGC that encodes cuniculene. Similarly, although not identified in its SPE extracts, *A. muelleri* possessed a singleton NRPS cluster with 100% similarity to that encoding for anabaenopeptin NZ857 (nostamide A), a highly toxic compound known for its inhibitory activity of proteases, phosphatases, and carboxypeptidases [67]. These may be examples of ‘silent’ SM-BGCs, a hypothesis that could be explored in the future by testing different culture conditions and extraction methods. Moreover, the anabaenopeptin NZ857 SM-BGC of *A. muelleri* may have been acquired by horizontal gene transfer, since anabaenopeptins are typically found in *Cyanobacteria* [67,68].

### 3.6. Metabolomics Analysis of Aquimarina Extracts Highlights Phylogenetic Relationships

Metabolomic and functional genomic information of the nine *Aquimarina* strains, followed similar patterns and were overall congruent with the strain taxonomy. For example, 16S rRNA gene homology and genome-wide average nucleotide identity (ANI) values indicate that Aq107 is closely related with *A*. *latercula*, and multivariate analyses demonstrated that both strains share similar genomic and metabolomic profiles. Likewise, Aq349, EL33 and EL43, which share *A. megaterium* as their closest type species, also displayed highly similar functional gene and metabolite profiles.

*A. spongiae* displayed the most distinct metabolomic profile. There was no previous indication that the metabolome of this species would significantly diverge from the remaining strains surveyed in this study, particularly from *A. latercula* and Aq107, with which *A. spongiae* formed a consistent functional group based on Clusters of Orthologous Groups of protein (COG) annotations in a previous survey [24]. However, in the Pfam-based genomics ordination of this study, *A. spongiae* was indeed somewhat separated from the other two strains.

Significant similarities were found between Aq135 and *A*. *muelleri,* which were overall the most active strains in the antimicrobial assays and clustered together in the metabolomics and genomics-based ordination diagrams. Moreover, in the metabolic networks, some unidentified clusters exclusively formed by Aq135 and *A. muelleri* nodes were present, indicating that they may be producing similar, potentially novel metabolites, in addition to a plethora of strain-specific compounds. Both strains belong to the same functional genome group (Group 1), previously reported by our team in a broad comparative genomics study of this bacterial genus [24]. However, Aq135 and *A*. *muelleri* genomes share ANI values of only 77.88% and 16S rRNA gene sequence similarity of 95.6% [24], suggesting that strain Aq135 likely represents a novel and yet-to-be-described species within the genus.

### 3.7. Long-Read Sequencing of Aquimarina Genomes Reveals Full Biosynthetic Potential

SM-BGCs are often found in the prokaryotic genome’s flexible (adaptive) part. In addition, their usually large size, the presence of repeat sequences and differential nucleotide usage of SM-BGCs (in comparison with the rest of the genome, especially when SM-BGCs have been subjected to horizontal gene transfer events), make their correct and complete assembly challenging, especially when short-read sequencing technologies, such as Illumina, are employed in genome sequencing [69,70]. Therefore, large SM-BGCs, such as the ones encoding NRPS and PKS, are often incomplete, complicating structure predictions for unknown molecules [71]. Genome fragmentation was particularly problematic for *A. muelleri,* whose genome, sequenced with Illumina technology and assembled thereafter into 107 contigs, had the higher percentage of incomplete SM-BGCs (61.9%). Quite likely, these SM-BGCs are split between contigs, artificially increasing the SM-BGC count in the *A. muelleri* genome assembly. Here, using a long-read sequencing technology would probably result in a smaller number of total SM-BGCs. In this study, resequencing of the *Aquimarina* strains from our in-house culture collection (Aq78, Aq107, Aq135, Aq349 and EL43) with the long-read sequencing PacBio technology led to the recovery of 100% complete SM-BGCs. Compared with the previous respective Illumina genome assemblies, the PacBio assemblies resulted in a much smaller number of contigs (1–3) of much greater length, and fragmented SM-BGCs were no longer a problem, opening doors for future structure elucidation and metabologenomics studies with these *Aquimarina* strains.

## 4. Materials and Methods

### 4.1. Exploring Abundance Distributions of Aquimarina spp. in the Marine Environment

The latest dataset released by the Sponge Microbiome Project [25] was used in this study to explore the relative abundance of *Aquimarina* spp. in marine settings, specifically in marine sponges (*n* = 3569 specimens) and surrounding habitats (seawater (*n* = 370), marine sediments (*n* = 65) and other environments, such as algae and biofilms (*n* = 29)). Briefly, this dataset comprises the taxonomic assignment of operational taxonomic units (OTUs, at 97% sequence identity), inferred from large-scale amplicon sequencing of the V4 region of the 16S rRNA gene from metagenomic DNA extracted from 4032 samples, using standardized procedures defined in [72]. In the present study, samples with less than 10,000 reads were removed to prevent acute skewness of estimated relative abundances, resulting in a final count of 3413 metagenomic DNA samples surveyed here for the presence of *Aquimarina* OTUs. Then, the dataset was filtered for samples that had at least one OTU taxonomically assigned to the genus *Aquimarina* by SILVA [73], Greengenes [74] or RDP [27]. This led to a total of 985 metagenomic DNA samples and 95 *Aquimarina* OTUs detected across the data. Since the latest taxonomical classification of these OTUs was performed in 2017, reclassification was carried out in this study with the RDP naïve Bayesian Classifier [75] v2.11, using RDP 16S rRNA gene training set number 18 (07/2020). Data visualization was achieved with Python packages matplotlib (v3.3.2) and seaborn (v0.11.0).

Phylogenetic reconstruction of the 95 OTUs retrieved above and the 16S rRNA gene sequences from all *Aquimarina* type strains and the nine isolates used here for bioactivity screenings, metabolomics and genomics was performed using the MEGAX v10.2.4 software package [76]. First, the sequences were aligned with MUSCLE [77] and, afterward, the Hasegawa-Kishino-Yano model [78] was inferred as the most suitable evolutionary model. Using the Maximum Likelihood method and the referred evolutionary model, phylogenetic tests with 1000 bootstrap repetitions were performed. The tree with the highest log likelihood (−1504.85) was selected. Initial tree(s) for the heuristic search were automatically obtained by applying Neighbor-Joining and BioNJ algorithms to a matrix of pairwise distances estimated using the Maximum Composite Likelihood (MCL) approach and then selecting the topology with a superior log-likelihood value. A discrete Gamma distribution was used to model evolutionary rate differences among sites (five categories (+G, parameter = 0.2995)). The analysis encompassed 148 nucleotide sequences, and all positions containing gaps and missing data were eliminated (complete deletion), leading to a total of 92 positions in the final dataset. Phylogenetic tree graphical visualization and styling were conducted in iTOL v4 [79].

### 4.2. Strains and Cultivation Conditions

#### 4.2.1. Aquimarina Strains

Nine *Aquimarina* strains were used in this study for antimicrobial activity bioassays and comparative genomics and metabolomics: six strains from an in-house culture collection of isolates of the marine sponges *Sarcotragus spinosulus* (Aq78, Aq107 and Aq349) and *Ircinia variabilis* (Aq135) [20] and of the octocoral *Eunicella labiata* (EL33 and EL43) [4] and three strains purchased from DSMZ, comprising the type strains of the species *A. muelleri* (DSM 19832) [9], *A. spongiae* (DSM 22623) [10] and *A. latercula* (DSM 2041) [26]. Bacterial cell purity was routinely assessed by streaking the isolates on 1:2 diluted marine agar (MA) plates. Additional information on these strains can be found in Table S2.

The choice of the nine strains was based on our previous study [24], where *Aquimarina* genomes were divided into three groups regarding phylogenomic and functional relationships. The nine strains represent all groups, with at least two isolates per group: group 1–*Aquimarina* sp. Aq135 and *Aquimarina muelleri* DSM 19832; group 2–*Aquimarina* sp. Aq349, *Aquimarina* sp. EL33, *Aquimarina* sp. EL43 and *Aquimarina* sp. Aq78 and group 3–*Aquimarina* sp. Aq107, *Aquimarina spongiae* DSM 22623 and *Aquimarina latercula* DSM 2041.

#### 4.2.2. Test Strains Used in Antimicrobial Assays

Antimicrobial activity assays were performed against a diversified panel of human pathogens and marine bacteria, whose general characteristics are presented in Tables S3 and S4, respectively. The panel of human pathogens included the Gram-positive bacteria *Staphylococcus aureus* 209 (ATCC 6538, DSM 799) and methicillin-resistant *Staphylococcus aureus* MRSA JE2 (NR-46543, B.E.I Resources–Part of ATCC); the Gram-negative bacteria *Pseudomonas aeruginosa* PAO1 (DSM 19880), *Escherichia coli* Seattle 1946 (ATCC 25922, DSM 1103) and *Salmonella enterica* subsp. *enterica* serovar Typhimurium SL1344 (DSM 24522) (heterotypic synonym *S. typhimurium*) and the fungal pathogens *Candida albicans* SC5314 (ATCC MYA-2876) and *Candida glabrata* KCHr606 (the latter originating from Chiba University, Japan [80]). The panel of marine bacteria was composed of 11 strains from an in-house culture collection of isolates, including eight *Vibrio* sp. strains [4], one *Micrococcus* sp. [20], one *Pseudovibrio* sp. [20] and one *Roseibium album* [4] strain. The closest reference (type) strain of each marine bacterium was identified with the RDP SeqMatch tool [27] (v3, RDP release 11.6). Additional information on these strains can be found in Table S4.

The strains were grown in appropriate liquid media as follows: Tryptic Soy Broth (TSB) for the Gram-positive human-pathogenic bacteria; Luria–Bertani Broth (LB) for the Gram-negative human-pathogenic bacteria; yeast extract-peptone-dextrose (YPD: 20 g/L glucose, 20 g/L peptone and 10 g/L yeast extract) or RPMI 1640-2% glucose medium at pH 7.0 (10,4 g/L RPMI 1640 (Sigma, Darmstadt, Germany), 34,5 g/L MOPS (Sigma, Darmstadt) and 18 g/L glucose) for *Candida* spp. [81]; Marine Broth (MB; Roth, Karlsruhe, Germany) diluted 1:2 in sterile artificial seawater (ASW: 23.38 g/L NaCl, 2.41 g/L MgSO_4_ × 7H_2_O, 1.90 g/L MgCl_2_ × 6H_2_O, 1.11 g/L CaCl_2_ × 2H_2_O, 0.75 g/L KCl and 0.17 g/LNaHCO_3_ [4]) for marine bacteria. The incubation temperature was 37 °C for all human-pathogenic bacteria and 30 °C for fungal pathogens and room temperature (RT) for marine bacteria.

### 4.3. Cross-Streak Assays

The cross-streak assay is an antimicrobial activity screening technique that delivers qualitative or semi-quantitative results on the inhibitory activities of a certain prokaryote isolate against a given test strain (usually also a prokaryote or a yeast) [28]. Fifteen microliters of *Aquimarina* spp. grown in MB (approximately 48 h of incubation at RT) were spread on 1.5% agar plates as a one-cm-wide line dividing the plate into two equal-sized halves (see examples in Figure S3a). The medium content of the agar plates varied according to the test microorganisms used: Mueller–Hinton agar (MHA) prepared with 3% ASW was used for marine bacteria, MHA prepared with 2% ASW for human-pathogenic bacteria and full-strength MA supplemented with 1% (*v*/*v*) of glucose for *Candida* strains. Preliminary assays with a variety of culture media showed that the above-mentioned media compositions enabled adequate growth of both *Aquimarina* strains and respective test strains. After an incubation period of five days at RT, five microliters of overnight-grown liquid cell cultures of the test strains were placed close to the *Aquimarina* central line, ensuring the absence of contact between the different strains. For homogeneous seeding, the test strain was streaked perpendicular to the central line (Figure S3a,b) with an inoculation loop, first toward the border of the plate and subsequently inwards, for a total of five streaks. For the cross-streak assay with *Candida*, the OD_600 nm_ of an overnight grown culture of the test strains was first measured and OD_600 nm_ adjusted to 0.1. Then, after additional incubation for 6–7 h, ODs were again adjusted to 0.1 before inoculation on the plates. After further incubation (24 h at 37 °C for human-pathogenic bacteria; 48 h at 30 °C for *Candida* spp.; 48 h at RT for marine bacteria) of the cross-streak plates, the overall growth of test strains and the size of inhibition zones was evaluated. All experiments were performed at least in duplicate for each *Aquimarina*-test strain pair. Agar plates inoculated only with the test strains were used as negative controls. Inhibitions were visualized and ranked as: (−) negative (normal growth of test strains, equal to controls); (+/−) weak (ca. 25% growth reduction compared with controls); (+) moderate (ca. 50% growth reduction); (++) strong (ca. 75% growth reduction); (+++) complete (no growth of the test strain) (Figure S3b).

### 4.4. Preparation of Extracellular Metabolite (Crude) Extracts from Aquimarina Strains

For the preparation of *Aquimarina* spp. crude extracts, well-grown pre-inocula (1.2 mL) of each strain were inoculated into 120 mL of 1:2 diluted MB and grown at 24 °C in an incubator (Labtron, Camberley, UK) with 120 rpm orbital shaking for two days. Afterward, cultures were centrifuged at 4 °C and 10,000 rcf for 35 min. Culture supernatants were collected and subjected to solid-phase extraction (SPE) following a similar procedure as described in [82]. Briefly, HLB Plus cartridges (Oasis, Waters, Milford, MA, USA) were attached to an SPE Visiprep 12 port vacuum manifold (Supelco, Sigma-Aldrich, Darmstadt, Germany) and cartridge activation was performed with 6 mL of 100% LC-MS-grade methanol (LiChrosolv^®^, VWR, Radnor, PA, USA) and washed with 6 mL GC-MS-grade (SupraSolv^®^, Merck, Darmstadt) water. The entire sample volume was passed first through an ISOLUTE^®^ depth filter (Biotage, Uppsala, Sweden) and then through the activated HLB cartridge, at an approximate flow rate of 1 mL/min with the help of a vacuum pump. Finally, after washing the cartridge with GC-MS-grade water, each sample was eluted with 6 mL LC-MS-grade methanol. The eluates were then evaporated in a gentle flux of nitrogen gas, reconstituted in 500 μL of methanol-water (50:50 *v*/*v*) and stored at −20 °C in glass vials until further use in broth microdilution assays and metabolome profiling, as explained below.

### 4.5. Broth Microdilution Assays

*Aquimarina* extracellular metabolite extracts prepared as described above were tested for antimicrobial activity using the broth microdilution method M07 as earlier described [83,84] in 96-well microplates (flat bottom; Sarstedt, Nümbrecht, Germany). Each *Aquimarina* extract was tested on each test strain at least twice. After overnight incubation of the test strains in the appropriate liquid medium (see Section 4.2.2 for details), pre-inoculum concentration was adjusted to the equivalent of 10^6^ CFU mL^−1^ in Mueller-Hinton broth (MHB) for bacterial human pathogens, double-strength RPMI 1640 medium [81] for assays involving *Candida* pathogens and MB when test strains were marine bacteria. One hundred microliters of cell culture of each test strain per microplate well was applied. Extracts were then serially diluted to reach final extract concentrations of 10%, 5%, 2,50%, 1,25%, 0,63%, 0,31%, 0,16%, 0,08% and 0,04% (*v*/*v*), respectively, in a total assay volume of 200 μL per well. Control wells only containing media and the test strain were prepared. In addition, methanol-water (50:50 *v*/*v*) only controls were tested at similar concentrations as the *Aquimarina* extracts to verify that the solvent did not affect growth of the test strains. The 96-well microplate was incubated without shaking for 24 h at 30 °C for all human pathogens and for 48 h at 24 °C for marine bacteria. Thereafter, ODs were measured at 600 nm using a microplate reader (SPECTROstar Nano, BMGLabtech, Ortenberg, Germany). The percentage of inhibition of test strain growth by each extract was calculated as follows: $\frac{OD\;control-OD\;extract}{OD\;control}\times 100$. Average inhibition values and their respective standard deviations were calculated. *Aquimarina* spp. crude extracts typically provoked the strongest growth inhibition at the highest (10% *v*/*v*) concentration tested, wherefore all results shown in this study correspond to 10% *v*/*v* concentration of crude extracts.

*Aquimarina* extracts that showed strong inhibition were subjected to further broth microdilution assays where the growth behavior of selected test strains was closely monitored over time [85]. Here, optical density (OD_600 nm_) was measured every 30 min on a FilterMax F5 microplate reader (Molecular Devices, San Jose, CA, USA) for a period of 24 h (human-pathogenic bacteria) or 48 h (marine bacteria) and growth curves in the presence of 10% (*v*/*v*) *Aquimarina* extracts, compared with controls, were generated, with at least two replicates per test strain. All conditions described above were maintained in these assays except that low-intensity shaking was introduced before each measurement.

### 4.6. Metabolomic Analyses of Aquimarina spp.

#### 4.6.1. UPLC-HR-MS/MS Profiling of Aquimarina Extracts

The chemical profiles of *Aquimarina* crude (SPE) extracts (three independent replicates per strain) were analyzed by liquid chromatography-high resolution mass spectrometry (LC-HR-MS) following similar procedures as described in [86]. Analyses were performed on a Thermo Scientific™ UltiMate™ 3000 UHPLC, coupled to an Orbitrap Elite (Thermo Fisher Scientific, Waltham, MA, USA) mass spectrometer with a Heated Electro-Spray Ionization source (HESI-II; Thermo Scientific). This hybrid Ion Trap-Orbitrap system enables simultaneous high-resolution and tandem-MS, with high detection power of low concentration metabolites across wide mass ranges. Five microliters of each extract (diluted 1:10 in 100% LC-MS grade methanol) were injected and separated on a Thermo Scientific Accucore RP-18 column (2.1 × 100 mm, 2.6 µm) in a 40 min run. A binary mobile phase consisting of ultra-pure LC-MS grade water (A) and LC-MS grade acetonitrile (B), both containing 0.1% formic acid, was used. The gradient (in *v*/*v* %) started with 100% of A during 2 min. The ratio of B/A increased linearly to 30% B in 13 min, then to 100% B in 16 min, and then stayed at 100% B for 4 min. The mobile phase then returned to 100% of A in 1 min and the column was stabilized at 100% of A for 4 min before the next run. Separation was performed at a flow of 0.3 mL/min. Data were acquired under positive and negative polarity (in separate runs) using the following parameters: spray voltage, 3.8 kV; sheath gas, 40 arbitrary units; auxiliary gas, five arbitrary units; heater temperature, 300 °C; capillary temperature, 350 °C; S-Lenses RF level, 64.9%. Scan range was 100–1500 m/z. The samples were analyzed in data-dependent mode by selecting the three most intense ions under dynamic exclusion and collision-induced dissociation (CID) activation. MS/MS fragmentation was achieved with a rising collision energy of 35 keV in an isolation window of 2. The minimum signal required for ddMS^2^ triggering was 1000. LC-MS data acquisition and analysis were performed using Xcalibur v4.1 Qual Browser (Thermo Scientific). Before running the samples, a solvent control, consisting of methanol-water 50:50 *v*/*v* % (the same solvent that was used to reconstitute all SPE extracts after extraction and evaporation), was run as well as a culture medium control (i.e., 1:2 diluted MB extracted with an HLB cartridge and treated in the same way as all *Aquimarina* samples). Intercalated with the remaining runs, a quality control, composed of a mixture of equal-volumed aliquots from all *Aquimarina* SPE extracts, was run four times.

#### 4.6.2. Metabolomic Data Processing and Molecular Network Analyses

Acquired spectra were converted to the open-access mzXML format with the ProteoWizard tool msConvertGUI v3.0.21141 [87] in centroid mode and uploaded through WinSCP v5.19.1 to the GNPS [29] website, along with a metadata description file of the samples. Classical molecular networking (v28.2) [29] was performed on GNPS as follows. First, all MS/MS fragment ions within +/− 17 Da of the precursor *m*/*z* were removed and MS/MS spectra were window filtered by choosing only the top six fragment ions in the +/− 50 Da window. Precursor and fragment ion tolerances were set to 0.01 Da. After network creation, edges with a cosine score below 0.7 and less than six matched peaks were removed. In addition, all edges between two nodes had to appear on each other’s top 10 most similar nodes or were eliminated. To comply with a maximum molecular family size threshold of 100, the lowest-scoring edges were removed from molecular families until the molecular family size was below this value. Spectra in the final network were further searched against GNPS spectral libraries and these spectra were treated in the same manner as the input data. To improve classical molecular network annotation, additional tools available at GNPS were used with default settings: DEREPLICATOR [88], DEREPLICATOR VarQuest [89], DEREPLICATOR+ [90] and MS2LDA [91]. The merging of these additional analyses with the previously obtained molecular network was performed with the MolNetEnhancer tool [30]. Network visualization was performed with Cytoscape 3.8.2 [92] and the AutoAnnotate app [93] was used to label clusters with the ‘CF_Dparent’ ClassyFire class annotation [65] from the MolNetEnhancer output.

In addition to classical molecular networking, we also performed feature-based molecular networking (FBMN) [31] using an integrated metabolomic workflow that includes MS-DIAL v4.70 [94], for spectral deconvolution and data alignment, MS-FINDER v3.52 [95,96], for peak annotation, and MS-CleanR [97], for feature filtration. First, mass spectrometry data (.raw files) were submitted to peak picking, alignment and deconvolution in MS-DIAL software using the following parameters: MS^1^ and MS^2^ tolerance of 0.01 and 0.025, respectively; 10,000 amplitude minimum peak width; 0.05 Da mass slice width; linear-weighted moving average smoothing method using three scans and peak width of five scans; sigma window value for deconvolution of 0.5; 0.2 min; 0.015 Da tolerance for peak alignment. This process was accompanied by peak annotation by MS-FINDER [95,96] using a MS-DIAL metabolomics MSP spectral kit that combines several publicly available MS/MS databases in positive and negative ionization polarity (last edited on 13 April 2021). Afterward, MS-DIAL resulting aligned peak lists were introduced into MS-CleanR [97] to perform feature filtration. First, MS-CleanR removes noise signals by applying generic filters. Here, a maximum relative standard deviation (RSD) of 40 and a relative mass defect (RMD) minimum of 50 and maximum of 3000 were used. In a second step, each feature is clustered based on the MS-DIAL peak character estimation algorithm, followed by parental signal extraction using multi-level optimization of the modularity algorithm. Finally, positive, and negative ionization modes are combined and adduct relationships corrected accordingly. The resulting “cleaned-up” feature list (see Table S5) was used to perform multivariate analysis on the metabolomics profiles of *Aquimarina* spp.

### 4.7. PacBio Genome Sequencing of Aquimarina Strains

The nine *Aquimarina* strains in study possess publicly available genome assemblies obtained from Illumina sequencing reads (Table S2). However, some genome sequences are quite fragmented and, consequently, several SM-BGCs remain incomplete. To improve overall genome sequence quality, *Aquimarina* strains Aq78, Aq107, Aq135, Aq349 and EL43 from our in-house culture collection were re-sequenced with PacBio sequencing technology (Pacific Biosciences Inc., Menlo Park, CA, USA). All PacBio assemblies are original contributions of this study except for strain Aq135, whose PacBio-sequenced genome was already published in Dieterich et al., 2022 [23]. Briefly, high-molecular weight genomic DNA was extracted with the Wizard Genomic DNA purification kit (Promega, Madison, WI, USA) according to the manufacturer’s instructions from cultures grown for two days at 24 ºC in MB. DNA quality and concentration were each assessed with a NanoDrop^TM^ 2000 spectrophotometer (Thermo Fisher Scientific, Waltham, MA, USA) and a Qubit^®^ 4.0 fluorometer (Thermo Fisher Scientific) with the dsDNA HS Assay Kit (Invitrogen, Waltham, MA, USA). A clean-up step was performed with the DNeasy Power Cleanup Kit (QIAGEN, Hilden, Germany). Genomic DNA samples were then shipped to MR DNA (Shallowater, TX, USA), where DNA was sheared in a Covaris G-tube (Covaris Inc., Woburn, MA, USA) and resulting fragment sizes were assessed via gel electrophoresis (E-Gel SizeSelect 2% Agarose Gel; Invitrogen, Waltham, MA, USA). Fragments from 6 kb to 10 kb were selected with the BluePippin automated size-selection instrument (Sage Science, Beverly, MA, USA) and the average sample size was verified with an Agilent 2100 Bioanalyzer (Agilent Technologies Inc., Santa Clara, CA, USA). Library preparation was performed with 100 to 200 ng of genomic DNA of each strain in a SMRTbell Express Template Prep Kit 2.0 (Pacific Biosciences Inc., Menlo Park, CA, USA). The library was sequenced using the 10-hour movie time on the PacBio Sequel System (Pacific Biosciences, Menlo Park, CA, USA). Afterward, genome de novo assembly was performed with the single-molecule real-time (SMRT) Analysis Hierarchical Genome Assembly Process (HGAP; SMRT Link 9.0.0). After genome assembly, genome statistics were gathered with the statswrapper tool from the BBTools suite v38.00 [98] (https://sourceforge.net/projects/bbmap/, accessed on 10 May 2022) and are available in Table S7.

### 4.8. Genome Annotation and SM-BGC Identification

Pfam profiles [99] were obtained for all genomes using our in-house, automated genome annotation pipeline MeLanGE, documented and available on GitHub (https://sandragodinhosilva.github.io/MeLanGE, accessed on 30 August 2021). Briefly, genomes were first annotated with Prokka v1.14.6 [100] to obtain GenBank (gbk) format and amino acid fasta files. Thereafter, proteins were queried, with the function hmmscan search (from HMMER v3.3.1), against a local database constructed with the latest Pfam-A.hmm (v35.0), containing hidden Markov model protein profiles. The best hit per ORF, above the cut-off of E 1e-5, was selected.

Identification of SM-BGCs on the nine genomes analyzed in this study was performed with the antiSMASH tool v6.0.1 [32] with default strictness (relaxed) and all extra features on. SM-BGC sequence similarity networks were calculated with BiG-SCAPE v.1.0 [33] in “hybrids” mode and groups of similar SM-BGCs were clustered into Gene Cluster Families (GCFs) at a 0.3 cut-off. The resulting SM-BGC network was visualized in Cytoscape v3.8.2 [92] using an unweighted “Prefuse Force Directed Layout”. SM-BGCs were considered known when they shared ≥70% similarity to a MIBiG reference SM-BGC, or, for cuniculene [22] and aquimarins [23], after manual inspection and comparison with the most recent literature.

### 4.9. Statistical Analyses and Data Visualization

Statistics and multivariate analyses were performed in an R (v4.0.4) environment. First, FBMN metabolomic feature list and Pfam profiles were Hellinger transformed (i.e., square root of relative abundance data) with the function *decostand* from the vegan v2.5.7 R package [101]. Then, a principal components analysis (PCA) was performed on transformed data with the *prcmp* function from stats R base package and visualized with the *fviz_pca_ind* function of the factoextra R package [102]. Permutational multivariate analysis of variance (PERMANOVA) tests were performed in Past v4.06 [103] to confirm the statistical significance of the clustering of *Aquimarina* into separate groups in the PCAs. Data manipulation and visualization were performed in Python v3.7.4 using the packages pandas v1.0.4, numpy v1.16.6, seaborn v0.9.0, matplotlib v3.0.3, scikit-learn v0.21.3 and in R v3.5.1 with tidyverse package dplyr v1.0.7 [104], ggplot v2 3.1.0 and vegan v2.5.7 [101] packages.

## 5. Conclusions

Our multidisciplinary study explored the frequency of occurrence, antimicrobial activities, and chemical space of the marine bacterial genus *Aquimarina*. We demonstrate that *Aquimarina* species are members of the rare microbial biosphere with relative abundances typically below 0.1% across diverse marine habitats. We find that *Aquimarina* species display widespread inhibitory activity against marine bacteria, particularly *Vibrio* spp., which could be relevant to aquaculture where vibriosis-related disease outbreaks are a notorious problem. Moreover, most *Aquimarina* strains showed noticeable inhibition of the human-pathogenic yeast *C*. *glabrata*, and crude extracts of *Aquimarina* sp. strains Aq135, Aq78 and *A. muelleri* also showed promising activities against Gram-positive human-pathogenic bacteria, such as MRSA, encouraging further marine drugs research.

This is the first study to deliver reproducible, reference metabolomics profiles for multiple *Aquimarina* species from all major functional groups of the genus. These profiles reveal a high level of congruency with the corresponding functional genomics profiles of the strains, whereby patterns are shaped by *Aquimarina* phylogeny. Annotation of metabolomics and SM-BGC networks suggests that multiple, novel secondary metabolites are yet to be uncovered from this genus, particularly type III polyketides and ribosomal and non-ribosomal peptides, including putative cyclic depsipeptide-related compounds. Taken together, our study emphasizes the relevance of *Aquimarina* spp. as a member of the microbial rare biosphere in the discovery of novel marine drug leads.

## Data Availability

PacBio assemblies of *Aquimarina* strains Aq78, Aq107, Aq349, and EL43, original contributions of this study, are available through accession numbers GCA_943416075, GCA_943733665, GCA_943373825 and GCA_943422735. The *Aquimarina* sp. Aq135 PacBio assembly, published in Dieterich et al., 2022, is available through accession number GCA_921010045.1. Remaining assembly accession numbers of Illumina assemblies are listed in Table S2. Metabolomics data created and presented in this study has been deposited to MassIVE under the accession numbers MSV000089550 (doi:10.25345/C5V698G86) for positive ionization polarity, and MSV000089549 (doi:10.25345/C5000043R) for negative ionization polarity. The ‘Sponge Microbiome Project’ dataset is deposited under NCBI BioProject ID PRJEB18736 and dataset files are available at http://gigadb.org/dataset/100332 (accessed on 10 May 2022).

## Supplementary Materials

The following supporting information can be downloaded at: https://www.mdpi.com/article/10.3390/md20070423/s1: Figure S1: Frequency of occurrence of *Aquimarina* OTUs across Sponge Microbiome Project (SMP) samples. Figure S2: Phylogenetic analysis of *Aquimarina* OTUs and reference strains. Figure S3: Example photographs of the antimicrobial assays performed in this study. Figure S4: LC-MS chromatograms of all (nine) *Aquimarina* isolates and replicates in positive ionization mode. Figure S5: LC-MS chromatograms of all (nine) *Aquimarina* isolates and replicates in negative ionization mode. Figure S6: SM-BGC count per *Aquimarina* genome analyzed in this study. Table S1: Sample origin, sponge taxonomic order and number of samples in which *Aquimarina* OTUs were found across the Sponge Microbiome Project (SMP) dataset; Table S2: Identification and general information of the *Aquimarina* strains analyzed in this study; Table S3: Identification and general information of the panel of human pathogens tested in this study; Table S4: Identification and general information the panel of marine bacterial isolates tested in this study; Table S5: ‘Cleaned-up’ feature list of feature-based molecular networking (FBMN) results of all *Aquimarina* extracts analyzed in this study; Table S6: Secondary metabolite biosynthetic gene clusters (SM-BGCs) of the nine *Aquimarina* strains studied here grouped into Gene Cluster Families (GCFs); Table S7: Genome assembly statistics of the nine *Aquimarina* strains analyzed in this study; Table S8: BiG-SCAPE sequence similarity network (SSN) pairwise results below raw distance 0.3 cut-off.
