# Supplementary material for: Insights into the Antimicrobial Activities and Metabolomes of Aquimarina (Flavobacteriaceae, Bacteroidetes) Species from the Rare Marine Biosphere
Source: Mar Drugs. 2022 Jun 28;20(7):423. doi: 10.3390/md20070423 (PMC9323603; doi:10.3390/md20070423)
Supplement: Supplementary file 1 [file marinedrugs-20-00423-s001.zip › SupplementaryFiles_Proof/Supplementary Figures_revised.pdf]

Supplementary Material

Insights into the antimicrobial activities and metabolomes of *Aquimarina* (*Flavobacteriaceae*, *Bacteroidetes*) species from the rare marine biosphere

Sandra G. Silva[1,2], Patrícia Paula[1,2], José P. da Silva[3], Dalila Mil-Homens[1,2], Miguel C. Teixeira[1,2], Arsénio M. Fialho[1,2], Rodrigo Costa[1,2,3]* and Tina Keller-Costa[1,2,*]

[1]iBB-Institute for Bioengineering and Biosciences and i4HB-Institute for Health and Bioeconomy, Instituto Superior Técnico, Av. Rovisco Pais, 1049-001 Lisbon, Portugal.

[2]Bioengeneering Department, Instituto Superior Técnico, Av. Rovisco Pais, 1049-001 Lisbon, Portugal.

[3]Centre of Marine Sciences (CCMAR/CIMAR LA), University of Algarve, Campus de Gambelas, 8005-139 Faro, Portugal.

* Correspondence: tinakellercosta@tecnico.ulisboa.pt, rodrigoscosta@tecnico.ulisboa.pt; Tel.: (+351) 21 841 3167, (+351) 21 841 7339.

Supplementary Figures

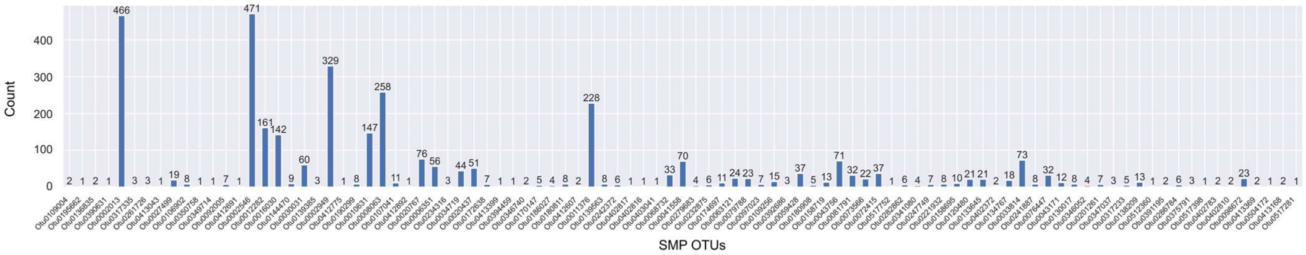

Figure S1. Frequency of occurrence of *Aquimarina* OTUs across Sponge Microbiome Project (SMP) samples. Values (counts) on top of each bar correspond to the number of samples in which each *Aquimarina* OTU was detected across the SMP dataset (from a total of 3413 SMP samples screened in this study). The 95 OTUs are ordered in descending order of median relative abundance across the dataset.

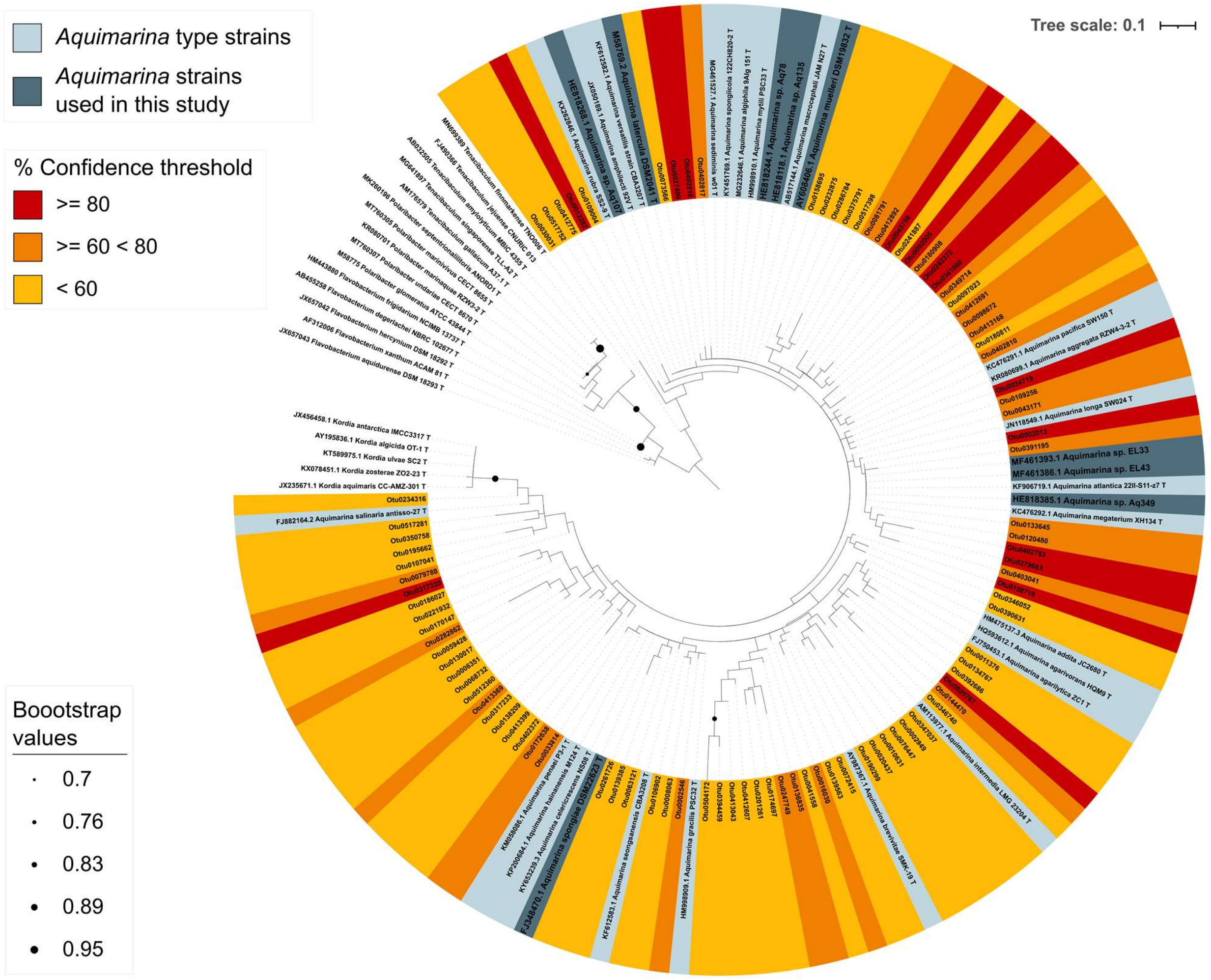

Figure S2. Phylogenetic analysis of *Aquimarina* OTUs and reference strains. 16S rRNA gene sequence phylogenetic tree constructed with the maximum likelihood method (Hasegawa-Kishino-Yano model). 16S rRNA gene sequences were aligned with MUSCLE. A total of 148 nucleotide sequences were employed to construct this tree, including all available 16S rRNA gene sequences of *Aquimarina* type strains (n = 24, light grey), 16S rRNA gene sequences of the nine *Aquimarina* strains used in this study (dark grey) and 16S rRNA gene sequences (V4 region) of SMP OTUs (n = 95). Twenty 16S rRNA gene sequences of members of phylogenetically close genera (*Kordia, Flavobacterium, Polaribacter*, *Tenacibaculum*) were used as outgroups. The complete deletion option was applied, leading to 92 nucleotide positions in the final dataset. SMP OTUs are coloured based on confidence values for classifying OTUs as *Aquimarina*, according to reclassification results obtained with the RDP SeqMatch tool. Bootstrap values (1000 repetitions) are shown, and the tree is drawn to scale.

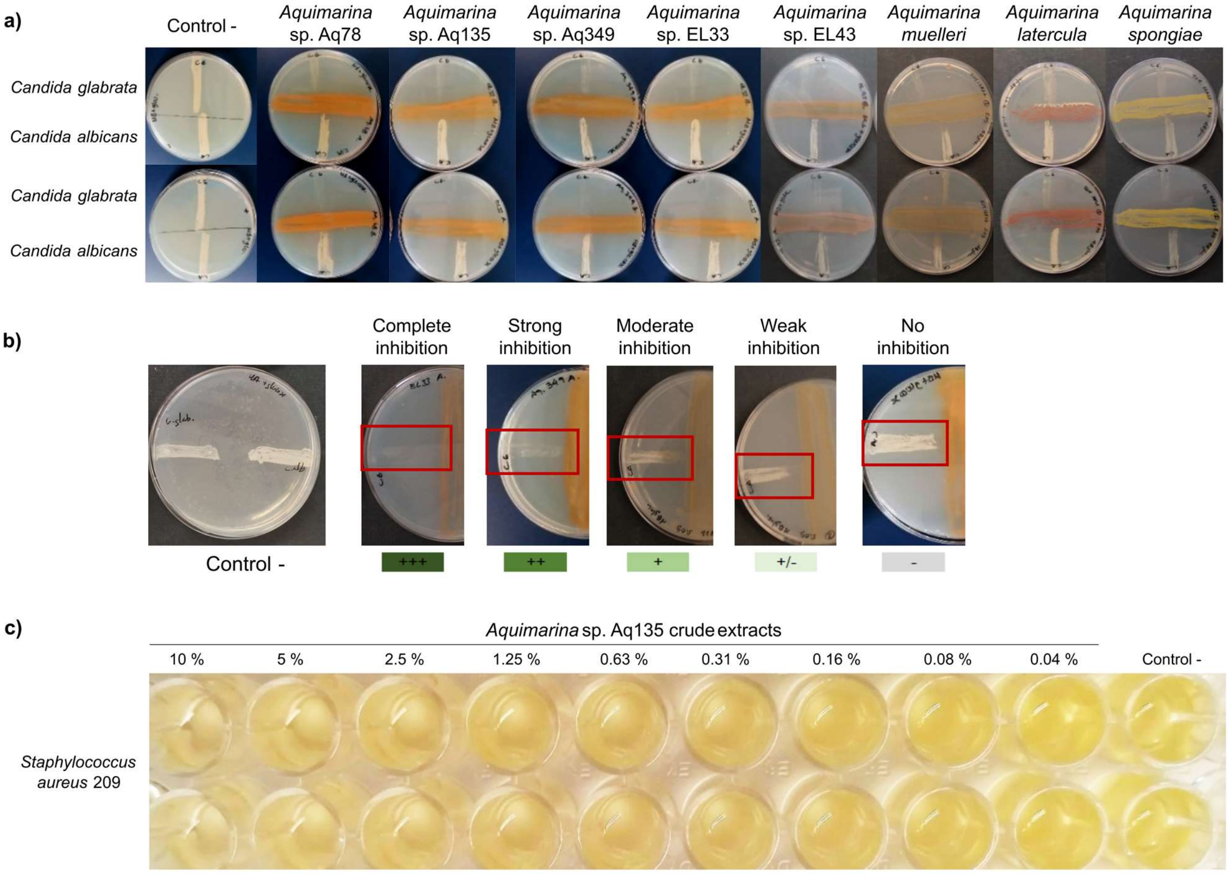

Figure S3. Example photographs of the antimicrobial assays performed in this study. (a) Example of a cross-streak plate assay result. Growth inhibition examination of *Candida glabrata* and *Candida albicans* when grown in the proximity of an *Aquimarina* spp. lane. Control (control -) plates show the two *Candida* strains grown without the presence of *Aquimarina*. All cross-streak assays were performed at least in duplicate. (b) Schematic representation of the levels of growth inhibition observed in the test strains and used to classify cross-streak assay results. (c) Example of a microdilution assay plate showing the growth of *Staphylococcus aureus* strain 209 (ATCC 6538) in the presence of various concentrations of *Aquimarina* sp. Aq135 crude extracts or in the absence of any extract (last column –control). Extracts were serially diluted from left to right.

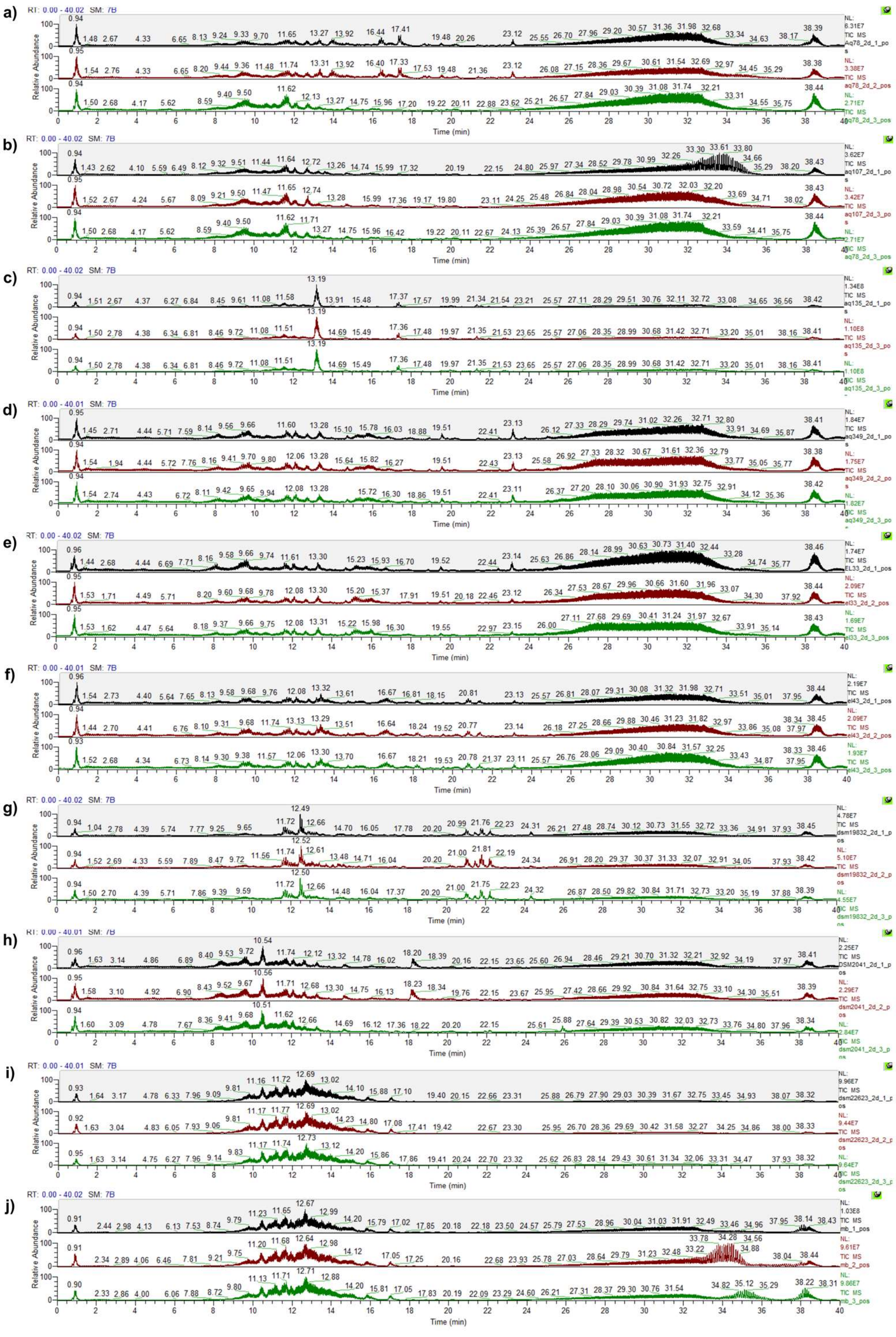

Figure S4. LC-MS chromatograms of all (nine) *Aquimarina* isolates and replicates in positive ionization mode. (a) *Aquimarina* sp. Aq78, (b) *Aquimarina* sp. Aq107, (c) *Aquimarina* sp. Aq135, (d) *Aquimarina* sp. Aq349, (e) *Aquimarina* sp. EL33, (f) *Aquimarina* sp. EL43, (g) *Aquimarina muelleri*, (h) *Aquimarina latercula*, (i) *Aquimarina spongiae*, (j) marine broth (MB) medium control. Three independent, biological replicates were analysed per strain.

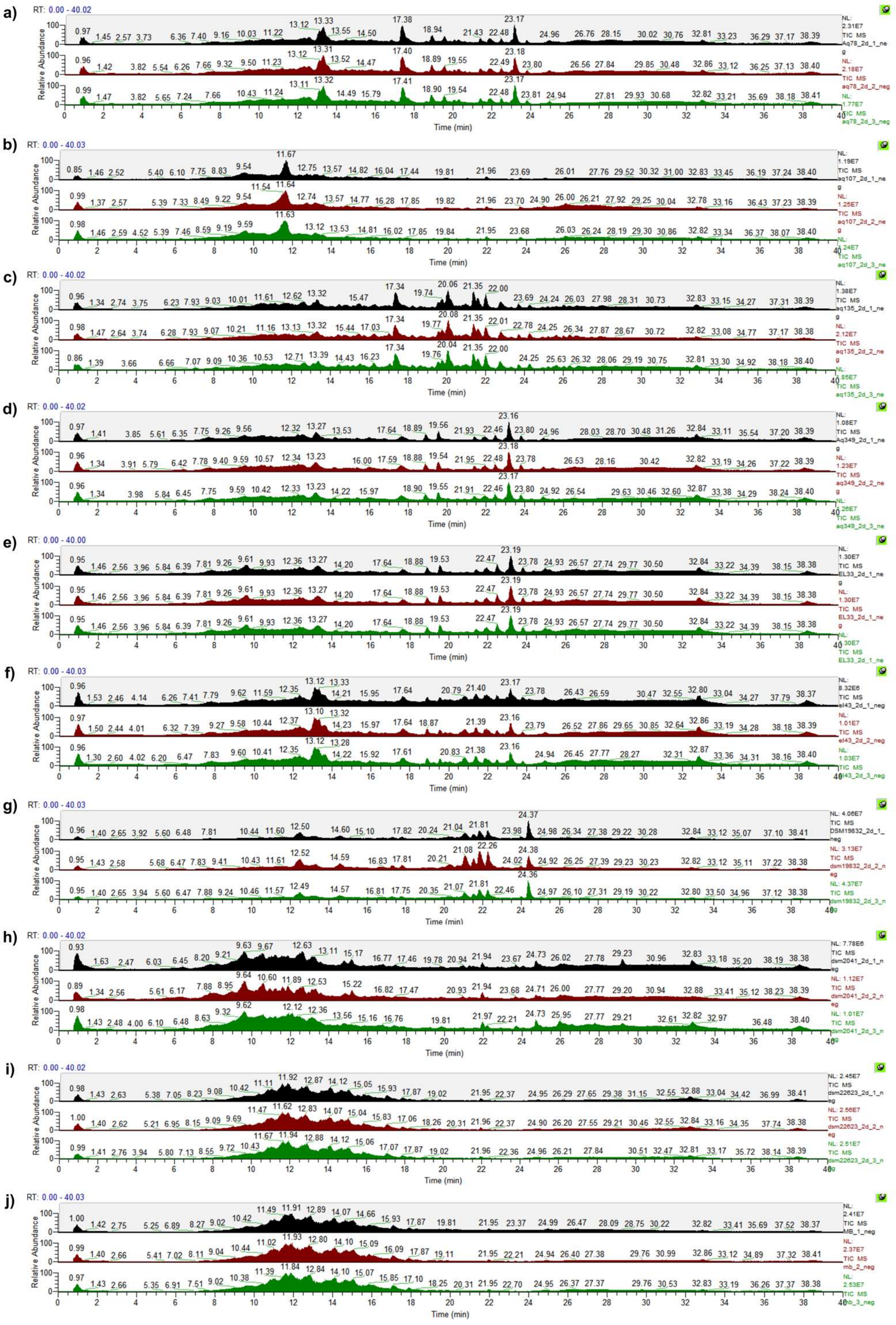

Figure S5. LC-MS chromatograms of all (nine) *Aquimarina* isolates and replicates in negative ionization mode. (a) *Aquimarina* sp. Aq78, (b) *Aquimarina* sp. Aq107, (c) *Aquimarina* sp. Aq135, (d) *Aquimarina* sp. Aq349, (e) *Aquimarina* sp. EL33, (f) *Aquimarina* sp. EL43, (g) *Aquimarina muelleri*, (h) *Aquimarina latercula*, (i) *Aquimarina spongiae*, (j) marine broth (MB) medium control. Three independent, biological replicates were analysed per strain.

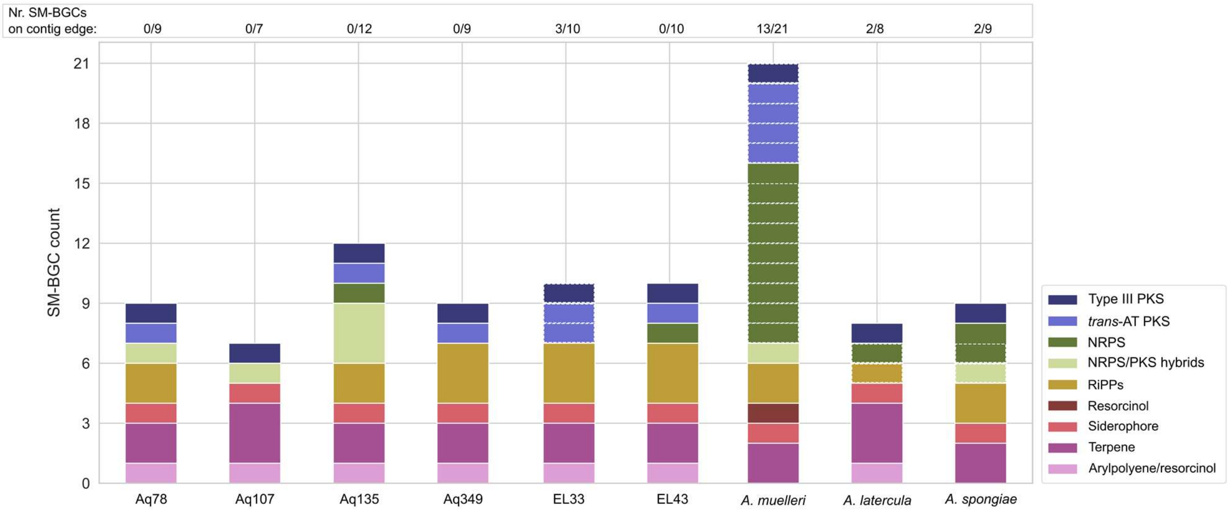

Figure S6. SM-BGC count per *Aquimarina* genome analysed in this study. SM-BGC classes are colour coded. SM-BGCs on the contig edge (incomplete SM-BGCs) are marked with a perforated white border, and the number of incomplete SM-BGCs is given above the stacked bars for each genome. Abbreviations: RiPPs - ribosomally synthesised and post-translationally modified peptides; NRPS - non-ribosomal peptide synthetase; PKS - polyketide synthase.
